# Prevalence of hypertension, diabetes, obesity, multimorbidity, and related risk factors among adult Gambians: a cross-sectional nationwide study

**DOI:** 10.1016/S2214-109X(23)00508-9

**Published:** 2024-01-01

**Authors:** Modou Jobe, Islay Mactaggart, Suzannah Bell, Min J Kim, Abba Hydara, Covadonga Bascaran, Modou Njai, Omar Badjie, Pablo Perel, Andrew M Prentice, Matthew J Burton

**Affiliations:** Medical Research Council Unit The Gambia at London School of Hygiene & Tropical Medicine, Fajara, Banjul, The Gambia; International Centre for Eye Health, London School of Hygiene & Tropical Medicine, London, UK; Moorfields Eye Hospital NHS Foundation Trust, London, UK; International Centre for Eye Health, London School of Hygiene & Tropical Medicine, London, UK; Sheikh Zayed Regional Eye Care Centre, Banjul, The Gambia; International Centre for Eye Health, London School of Hygiene & Tropical Medicine, London, UK; Directorate of Health Promotion & Education, Ministry of Health, Banjul, The Gambia; Department of Non-communicable Disease Epidemiology, London School of Hygiene & Tropical Medicine, London, UK; Medical Research Council Unit The Gambia at London School of Hygiene & Tropical Medicine, Fajara, Banjul, The Gambia; International Centre for Eye Health, London School of Hygiene & Tropical Medicine, London, UK; National Institute for Health Research Biomedical Research Centre for Ophthalmology, Moorfields Eye Hospital NHS Foundation Trust, London, UK

## Abstract

**Background:**

As countries progress through economic and demographic transition, chronic non-communicable diseases (NCDs) overtake a previous burden of infectious diseases. We investigated the prevalence of hypertension, diabetes, obesity, and multimorbidity in older adults in The Gambia.

**Methods:**

We embedded a survey on NCDs into the nationally representative 2019 Gambia National Eye Health Survey of adults aged 35 years or older. We measured anthropometrics, capillary blood glucose, and blood pressure together with sociodemographic information, personal and family health history, and information on smoking and alcohol consumption. Hypertension was defined as systolic blood pressure of 140 mmHg or more, diastolic blood pressure of 90 mmHg or more, or receiving treatment for hypertension. Diabetes was defined as fasting capillary blood glucose of 7 mmol/L or more, random blood glucose of 11·1mmol/L or more, or previous diagnosis or treatment for diabetes. Overweight was defined as BMI of 25–29·9 kg/m^2^ and obesity as 30 kg/m^2^ or more. Multimorbidity was defined as the coexistence of two or more conditions. We calculated weighted crude and adjusted estimates for each outcome by sex, residence, and selected sociodemographic factors.

**Findings:**

We analysed data from 9188 participants (5039 [54·8%] from urban areas, 6478 [70·5%] women). The prevalence of hypertension was 47·0%; 2259 (49·3%) women, 2052 (44·7%) men. The prevalence increased with age, increasing from 30% in those aged 35–45 years to over 75% in those aged 75 years and older. Overweight and obesity increased the odds of hypertension, and underweight reduced the odds. The prevalence of diabetes was 6·3% (322 [7·0%] women, 255 [5·6%] men), increasing from 3·8% in those aged 35–44 years to 9·1% in those aged 65–75 years, and then declining. Diabetes was much more common among urban residents, especially in women (peaking at 13% by age 65 years). Diabetes was strongly associated with BMI and wealth index. The prevalence of obesity was 12·0% and was notably higher in women than men (880 [20·2%] *vs* 170 [3·9%]). Multimorbidity was present in 932 (10·7%), and was more common in women than men (694 [15·9] *vs* 238 [5·5]). The prevalence of smoking was 9·7%; 5 (0·1%) women, 889 (19·3%) men. Alcohol consumption in the past year was negligible.

**Interpretation:**

We have documented high levels of NCDs and associated risk factors in Gambian adults. This presents a major stress on the country’s fragile health system that requires an urgent, concerted, and targeted mutisectoral strategy.

**Funding:**

The Queen Elizabeth Diamond Jubilee Trust and Wellcome Trust.

## Introduction

The Sustainable Development Goal target 3.4 is a 33% reduction in premature mortality from non-communicable diseases (NCDs).^1^ WHO adopted additional voluntary targets to reduce raised blood pressure by 25%, to halt the rise in diabetes and obesity, and to reduce tobacco use by 30% and harmful alcohol consumption by 10% by 2025.^2^ A crucial step to addressing premature mortality from NCDs in countries, such as The Gambia, requires mapping their prevalence at the population level to identify high risk groups for targeted primary and secondary prevention.

The Gambia is a low-income country in West Africa that, like others in the subregion, is faced with a double burden of communicable and non-communicable diseases.^3^ The country is undergoing substantial epidemiological, nutritional, and demographic transitions. These transitions, coupled with rapid unplanned urbanisation,^4^ will most likely drive further increases in NCDs if left unchecked. The country’s under-resourced health system was originally designed to manage infectious diseases. The country operates a three-tier health system, which is widely available to the population. The primary (eg, village and community clinics, and minor health facilities), secondary (eg, minor and major health centres, and regional hospitals) and tertiary (eg, general and teaching hospitals) levels of the national health system all provide care for NCDs at varying levels of quality.^5^ These facilities, especially those at the primary level, generally do not have adequate human and infrastructural resources for prevention and treatment of NCDs, which increasingly account for a high burden of long-term illness and early death.^6^

Hypertension, diabetes, and obesity are major NCDs leading to complications, such as ischaemic heart disease, stroke, chronic kidney diseases, and cancers.^7^ Their prevention and timely management should be a priority. This requires high-quality population-based data and up-to-date studies. The most recent of such studies in The Gambia was the 2010 WHO STEPS survey,^8^ which included a much younger population and was limited in investigating issues, such as clustering of comorbidities. The present study provides up-to-date data with a particular focus on middle-aged and older adults who are disproportionately affected by NCDs.^9^

We report a large, nationally representative, cross-sectional study to assess the prevalence of hypertension, diabetes, obesity, multimorbidity, and related risk factors in adults aged 35 years or older in The Gambia.

## Methods

### Study design and participants

We embedded a survey on NCDs into the nationally representative 2019 Gambia National Eye Health Survey of adults aged 35 years or older. The detailed methodology is described elsewhere.^9^ Briefly, between February and July, 2019, we used a multistage stratified cluster random sampling procedure. Clusters were the standard national census enumeration areas, used by The Gambia Bureau of Statistics in the 2013 Population and Housing Census. For the purposes of this survey, we divided the country into three broad historical regions (ie, western, central, and eastern). The three regions were stratified into urban and rural clusters according to The Gambia Bureau of Statistics definition (western: 43 rural and 173 urban; central: 44 rural and 12 urban; eastern: 71 rural and 17 urban).^4^ Clusters were selected within each stratum using probability proportionate to size sampling methods. In the selected cluster, enumerators from the Gambia Bureau of Statistics listed all eligible participants and then grouped them into segments of 30 participants. A segment was then selected at random. The study protocol was approved by the Joint MRC–Gambia Government Ethics Committee (SCC 1635) and the London School of Hygiene & Tropical Medicine Ethics Committee (ref 16172).

### Sample enumeration and data collection

The data were collected by four survey teams, each having one ophthalmologist, one optometrist or optometry technician, one senior ophthalmic medical assistant, one general nurse, and two enumerators. Study staff were trained on study procedures and the questionnaire was pretested in a random sample of the population. Consent was sought from the household head or key informant, and from each eligible household member. Household members were considered eligible if they were aged at least 35 years, had lived in the household for at least 6 months of the previous year, ate shared meals with other household members, and did not pay, nor were paid by, other household members.

All consenting eligible household members were invited to attend the survey screening at an identified central community location the following day, and asked not to have breakfast on the survey day until after they had been visited at home by the team nurse.

Enumerators visited each household within the segment door-to-door until 30 eligible participants had been recorded. If the total number of 30 eligible participants was exceeded within a household, the required number of participants needed was selected at random. If fewer than 30 eligible participants were identified within the segment, a second segment was randomly selected to complete the cluster. Individuals were recorded as non-responders when they were not available after two repeated visits.

On the day of the survey, the team first visited each household in the segment to take a fasting capillary glucose measurement (Accu-Chek Aviva, Roche Diagnostics, Mannheim, Germany; detection range of 0·6 mmol/L and 33·3 mmol/L) before inviting participants to a central location where breakfast was provided before the remaining assessments. Sociodemographic and clinical information was electronically captured using the Open Data Kit application.

The questionnaire gathered data on age, sex, highest level of education, ethnic group, marital status, occupation, family history of hypertension, alcohol consumption, smoking status, wealth status, previous diagnoses of diabetes and hypertension, and current medication use for diabetes and hypertension.

Height was measured with the participants standing fully erect against a stadiometer (Leicester Height Measure, Birmingham, UK), without footwear or headwear, with the measurement to the nearest 0·1 cm. Weight was measured to the nearest 10 g (Seca, Hamburg, Germany). BMI was calculated as weight (kg) divided by height squared (m^2^).

Blood pressure was measured with the participant seated after resting for at least 10 min and with their arm supported at the level of the heart and resting on a surface. Measurement was initially taken in each arm and then repeated in the arm with the higher reading with automated OMRON-Healthcare 10 Series blood pressure monitors (Omron, Kyoto, Japan). The blood pressure measurements were taken 5 min apart, and an average of the last two measures was recorded for analysis. The EquityTool, as previously reported, was used to calculate wealth status.^9,10^

### Definition of outcome variables and covariates

We defined hypertension as systolic blood pressure of 140 mmHg or more, or a diastolic blood pressure of 90 mmHg or more, or a participant report of receiving medication for hypertension. Diabetes was defined as elevated blood sugar level, categorised as a fasting blood glucose of 7·0 mmol/L or more, or random blood glucose of 11·1 mmol/L or more, or a previous diagnosis or participant report of receiving treatment for diabetes. Participants who were identified with elevated blood pressure or elevated blood glucose, or both, were referred to the nearest health facility for further review and management.

Participants were classified as underweight (<18·0 kg/m^2^), normal weight (18·0–24·9 kg/m^2^), overweight (25·0–29·9 kg/m^2^), and obese (≥30·0 kg/m^2^), on the basis of their calculated BMI. We defined multimorbidity as a co-occurrence of at least two conditions of hypertension, diabetes, and obesity in a participant.

Level of education was defined according to the highest level attained in either a conventional school or madrassa (ie, Arabic or Islamic school). Data on occupation were categorised (from self-report) as unemployed, manual, trades, professional, other, and retired or due to old age. Ethnicity was categorised on the basis of self-attribution. We recorded marital status as never married, currently married, widowed, or divorced. Alcohol use was defined as any self-report of alcohol consumption in the past 12 months. Smoking status was categorised, as reported by participants, as never a smoker, current smoker, or past smoker.

### Statistical analysis

Sample size was calculated to enable detection of disease prevalence as low as 0·5%, such as blindness with a 95% confidence level and a margin of error of 0·25%. Given that the samples were drawn from clusters with an average of 30 individuals, a design effect of 2·5 was applied, assuming that samples were moderately clustered with an intraclass correlation coefficient of 0·038. A 20% non-response or dropout rate was also factored in, resulting in the final sample size of 10 800. During data collection, we addressed the potential bias with missing data of the wealth quantile by reapproaching respondents in clusters that had more than 50% missing data. As a result, all clusters in our survey had a higher than 50% response rate. For the remaining missing data in clusters that had more than 30 participants and less than 50% missing data, we did an imputation on each of the 12 socioeconomic questions that make up the wealth quantile with the most frequently observed value in the same cluster. The rationale for this approach was that we expected people living in the same cluster to have similar levels of socioeconomic status. It should be noted that we did an imputation only on the socioeconomic status questions individually. Imputation was not done on any other variables in the study because we minimised missing data during the initial data collection stage. Sensitivity analysis (appendix 1 p 4) showed that the imputation did not result in any systematic difference in the overall prevalence of vision impairment as well by the wealth quintile. The approach to handling of missing data in the present study has been described in detail elsewhere^9^ and in appendix 1. We accounted for the multistage sampling survey design in the analysis. In the present analysis, we estimated the prevalence rates of hypertension, diabetes, obesity, multimorbidity, and other risk factors, such as smoking and alcohol consumption, stratified by sex and residence (urban *vs* rural). Prevalence estimates of groups whose 95% CIs did not overlap were regarded to be significantly different. Considering the disproportionate female population in our sample, sampling weights were applied according to the population distribution of the 2013 Gambia Population and Housing Census,^4^ to account for the difference in age, sex, cluster, and location.^9^ We used logistic regression to investigate the association between each outcome of interest and each potential explanatory variable, stratified by sex and residence. For the adjusted analysis, we used a conceptual framework in which we categorised risk factors for hypertension and diabetes into non-modifiable and contextual factors, assuming these influenced the modifiable factors (appendix 1 p 2). We therefore used this framework to determine factors for inclusion in adjusted models, with one model including non-modifiable and contextual factors (appendix 1 p 2) and fully adjusted model including modifiable factors in addition to non-modifiable and contextual factors. Stata software (version 17) was used for all statistical analysis.

### Role of the funding source

The funders of the study had no role in study design, data collection, data analysis, data interpretation, or writing of the report.

## Results

A total of 11 127 participants were enumerated nationwide of whom 9788 took part in the survey. After exclusion of 600 participants with either missing household data or incomplete individual data, we included 9188 participants in the present analysis. The crude (unweighted) sociodemographic characteristics of the participants were previously reported^9^ and are in appendix 1 (p 5). There were more participants from urban areas than from rural areas (5039 [54·8%] *vs* 4149 [45·2%]), and more women than men (6478 [70·5%] women *vs* 2710 [29·5%] men) in both urban and rural areas.

The mean age of the population, regardless of residence, was similar in men and women (table 1). Women from rural areas were least likely to be involved in professional jobs and had a lower level of education whereas men from urban areas were more likely to have attained a higher level of education. Rural residents were of lower socioeconomic status, with no rural residents in the fifth (richest) wealth quintile.

Prevalence of hypertension was 47·0% (95% CI 45·6–48·5); significantly higher in women and in urban areas (table 2; appendix 1 pp 6–7). The odds of hypertension did not differ by residence in men and women (table 3). There was a steep rise in prevalence of hypertension with age in the age groups 35–44 years, 45–54 years, 55–64 years, and 65–74 years before a plateau in the older age groups (figure; appendix 1 pp 6–7). The prevalence of hypertension was mostly similar between ethnic groups, but higher in the Sarahuleh. The odds of hypertension were significantly higher in individuals who were overweight (1·64 [95% CI 1·29–2·09] in men and 1·47 [1·28–1·69] in women) and in individuals who were obese (1·81 [1·18–2·77] in men and 2·58 [2·23–2·98] in women), and significantly lower in individuals who were underweight (0·63 [0·45–0·88] in men and 0·65 [0·49–0·85] in women), even after adjusting for covariates. In the fully adjusted model, neither smoking or wealth were associated with hypertension (table 3).

The nationwide prevalence of diabetes was 6·3% (95% CI 5·7–6·9); similar in men (5·6% [4·6–6·5]) and women (7·0% [6·3–7·7]). The prevalence of diabetes was higher among women from urban areas compared with their rural counterparts, whereas the prevalence was similar for men (table 2; appendix 1 pp 8–9) but adjustment for covariates eliminated all rural and urban differences (table 3). There was no difference in prevalence between age groups. Diabetes was more common in men who were overweight and in women who were obese. Men in the richest (fifth) quintile quintile had higher odds of diabetes compared with those in the poorest quintile (2·73 [1·02–7·31]) after adjusting for covariates. In women, there were lower odds of diabetes in those in the second (0·51 (0·31–0·84]), third (0·63 [0·42–0·93]), and fourth (0·61 [0·40–0·93]) wealth quintiles, respectively, compared with those in the first (poorest) quintile (table 3).

More than one-third of the study population (3201 [36·6%]) were either overweight (2151 [24·6%]) or obese (1050 [12·0%]). The combined rates of overweight and obesity were twice as high in women than men (48·1% *vs* 25·2%; table 2). Obesity rates were also significantly higher in women compared with men in the overall population ([20·2% [18·8–21·5] *vs* 3·9% [3·0–4·8]), and in both urban (25·8% [23·8–27·7] *vs* 4·5% [3·2–5·8]) and rural areas (12·3% [10·6–14·0] *vs* 3·3% [2·0–4·6]). Obesity was more common in women from urban areas than in rural areas, but there was no difference for men (figure C; table 2; appendix 1 pp 10–11). In women, the odds of obesity were higher in the age groups 45–54 years and 55–64 years, and lower in the older age groups (75–84 years and ≥85 years), compared with the youngest age group. There was no statistically significant association between obesity and wealth quintile or alcohol consumption (table 4). Men who smoke had lower odds of obesity (0·39 [0·19–0·82]) compared with lifetime abstainers and previous smokers.

Alcohol consumption was very low (101 [1·1%] of 9188) and similar in both sexes (table 2). The overall prevalence of smoking was 9·7% (897 of 9188), and almost exclusively in men. 889 (19·3%) of 4598 men were current smokers compared with five (0·1%) of 4590 women. 682 (14·8%) men and one (<0·1%) woman were previous smokers.

Of the 8706 participants with complete NCDs data, multimorbidity was present in 10·7% (9·9–11·5) and was more prevalent in women, affecting 19·7% (18·0–21·5) in urban areas and 10·6% (9·0–12·1) in rural areas. The corresponding prevalences in men were 5·8% (4·6–7·1) and 5·1% (3·8–6·4). Very few people had all three conditions (0·9% [0·7–1·1] overall). The most common combination was hypertension and obesity, which was present in 7·2% (6·6–7·9), 12·2% (11·1–13·3) in women and 2·2% (1·6–2·8) in men. The combination of hypertension and diabetes was present in 4·2% (3·7–4·6); higher in women (4·9% [4·3–5·5]) than men (3·5% [2·8–4·1]). Obesity and diabetes coexisted in only 1·3% (1·0–1·5); 2·2% (1·8–2·6) in women and 0·3% (0·1–0·6) in men (table 2; appendix 1 pp 12–13).

## Discussion

Our nationally representative survey recorded very high levels of hypertension, and concerning levels of obesity, diabetes, and multimorbidity in adults aged 35 years or older. Like many developing nations, The Gambia has made good progress in reducing the prevalence of many infectious diseases, and is on track to meet several health targets of the Sustainable Development Goals. Unfortunately, the pendulum is swinging from undernutrition to overnutrition, with its associated ill health.

There have been few nationally representative surveys to evaluate the burden of cardiovascular risk factors in The Gambia. Our study shows a higher burden of hypertension for similar age groups than the 2010 NCD WHO STEP survey,^8^ possibly reflecting the pace of urbanisation, and changes in dietary patterns and lifestyle are occurring in The Gambia. Our data are similar to recent reports from neighbouring countries, such as Sierra Leone^11^ and Senegal;^12^ although, the Senegal study included a younger population. A nationwide survey in Guinea found nearly two-thirds of adults aged 44–64 years had hypertension.^13^ The similar prevalence in urban–rural areas in our study was not consistent with the 2010 Gambia NCDs survey in which a significantly higher prevalence in rural areas was reported. This was in contrast with reports elsewhere in sub-Saharan Africa. A meta-analysis of 22 studies in West Africa showed lower odds of hypertension in rural locations.^14^ In The Gambia where nearly 60% of the population reside in urban areas,^15^ similar high dietary salt intake in urban and rural areas^16^ might explain the similar prevalence. We observed a weak association between hypertension and wealth status. A multicentre study from 12 low-income and middle-income sub-Saharan African countries shows that the burden of hypertension is highest in individuals in lowest wealth groups in low-income countries.^17^

The prevalence of diabetes in The Gambia appears to be increasing, especially in rural areas. Compared with a 1997 survey, the prevalence in urban areas remains similar (7·9% in men and 8·7% woman in 1997 *vs* 6·8% and 8·6%, respectively, in 2019 as of current study). However, there is a marked increase in the prevalence of diabetes in rural areas (2·2% in men and 0·8% in women in 1997 *vs* 4·3% and 4·8%, respectively, in 2019).^18^ This urban–rural difference in prevalence rates (possibly also applicable for obesity rates) could be due to higher availability and intake of sugars and processed foods in urban areas, and the higher physical activity levels in rural populations. A 2020 survey comparing contemporary prevalence estimates in Sierra Leone reported a diabetes prevalence of 3·5% in people over the age of 40 years.^11^ The NCD Risk Factor Collaboration projected, for 2014, a prevalence of diabetes of 9·4% for men and 7·9% for women,^19^ compared with 5·6% and 7·0%, respectively, in our survey. Our reported 2019 prevalence of 6·3% greatly exceeds the International Diabetes Federation’s Diabetes Atlas 2019 estimates of 1·6% for The Gambia.^20^ Although a useful source of information, the Diabetes Atlas has limitations, including extrapolation of data from countries with similar economy, language, and demography.

The high prevalence of obesity, especially in women, is consistent with the 2010 STEP survey in The Gambia.^21^ Again, these contemporary estimates show a large increase compared with data from the late 1990s (2·3% nationwide).^22^ Notwithstanding the complex causal drivers for the rise in prevalence in low-income and middle-income countries,^23^ biocultural factors in our setting appear to be key determinants behind the higher prevalence observed among women. In a study in neighbouring Senegal, middle-aged and older women were found to value being overweight or obese more than their younger counterparts,^24^ corroborating earlier findings in The Gambia, which additionally reported that women with an education tended to appreciate a small body size. Obesity in The Gambia is still commonly regarded as a sign of wealth, influence, and strength, especially among women.^25^

We are not aware of any previous studies on multimorbidity in The Gambia. Our data also shows high prevalence of NCDs multimorbidity in The Gambia, with women being disproportionately affected. Our study found a higher prevalence of multimorbidity than was found in Malawi (in a survey also including younger age groups), where the most common combination was also hypertension and obesity.^26^ A systematic review of multimorbidity in South Africa found prevalence of multimorbidity depended on the age groups included, ranging from 3% to 23% in studies including participants aged 15 years or older, whereas this was between 30% and 70% in those aged 50 years or older.^27^

Hypertension rates are strongly associated with age, increasing from 30% at 35–45 years to 78% at 75 years or older. Obesity, diabetes, and multimorbidity showed the lowest rates at the extremes of ages—ie, younger than 45 years and older than 75 years. Lower prevalence of hypertension in older age groups might represent either a cohort effect (older adults matured before the main effects of social and economic transition were felt) or a healthy survivor effect (ie, that people without hypertension might live longer).

Both hypertension and diabetes were strongly associated with obesity in the unadjusted analysis. The association is attenuated by adjustment for modifiable, non-modifiable, and contextual factors. Although the association of diabetes with obesity is strong, the odds ratios are much lower than in some White populations in the USA.^28^

Despite its robust design and large sample size, our study should be considered with some limitations. We included only adults aged 35 years or older and hence might not be generalisable to the younger population who account for most of the population. Substitution of missing socioeconomic data with the most frequently observed value, adopted in our approach, might result in artificial reduction in variability of socioeconomic position levels within a cluster. Additional factors that were not collected in the present study, such as physical activity level, salt intake, sugar consumption, and access to food and health care, would have increased understanding of the drivers of these NCDs. Furthermore, we used capillary glucose, which is not considered as the gold standard for the assessment of diabetes status. Although this has been shown to be reliable and even performing better than HBA1C in some cases,^29^ the estimates might be different from those obtained in clinical care. Finally, in estimating the burden of multimorbidity, we only included three conditions that will likely underestimate the true prevalence in The Gambia.

We have documented high prevalence of NCDs in The Gambia. Due to weak health systems in sub-Saharan Africa, hypertension and diabetes, and obesity and multimorbidity, generally lead to worse outcomes, including premature death. This high prevalence of NCDs and related risk factors presents a major, and likely growing, stress on fragile health systems, highlighting the need for a concerted multisectoral approach to NCDs.

## Supplementary Material

Supplementary appendix 1

Supplementary appendix 2

## Figures and Tables

**Figure:**
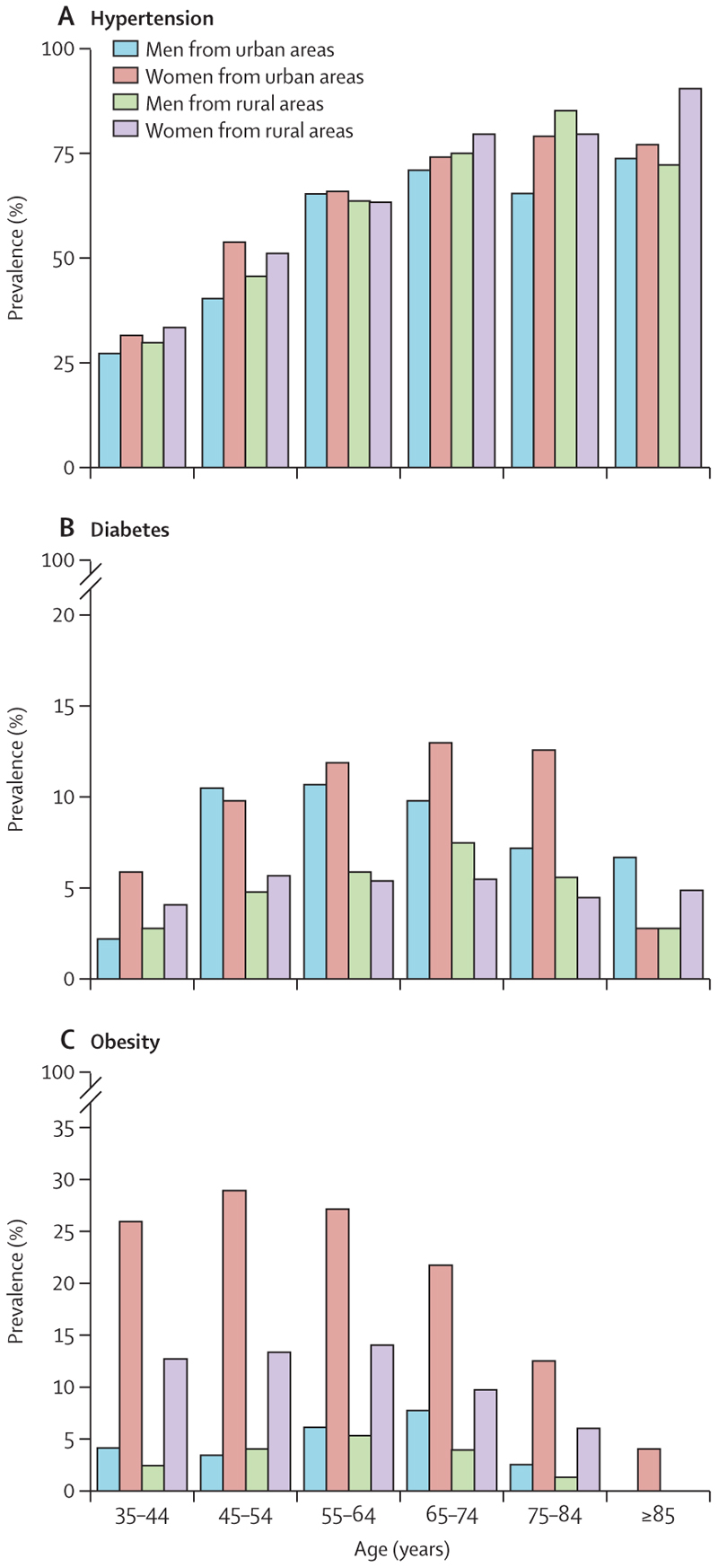
Prevalence of obesity, hypertension, and diabetes by age group among adults aged 35 years or older in The Gambia Prevalence rates weighted for age, sex, and cluster size.

**Table 1:** Age and sex-standardised sociodemographic characteristics of participants weighted for cluster size

	All (n=9188)	Men (n=4598)	Women (n=4590)	Urban areas (n=5039)			Rural areas (n=4149)	
				Men (n=2339)	Women (n=2700)		Men (n=2255)	Women (n=1894)
Age, years	49·5 (0·18)	49·7 (0·25)	49·3 (0·22)	50·0 (0·39)	49·3 (0·33)		49·3 (0·28)	49·4 (0·36)
35–44	3995 (43·5%)	1952 (42·5%)	2043 (44·5%)	988 (42·2%)	1190 (44·1%)		962 (42·7%)	855 (45·1%)
45–54	2462 (26·8%)	1259 (27·4%)	1203 (26·2%)	604 (25·8%)	728 (27·0%)		653 (29·0%)	476 (25·1%)
55–64	1350 (14·7%)	715 (15·6%)	635 (13·8%)	392 (16·8%)	388 (14·4%)		323 (14·3%)	248 (13·1%)
65–74	808 (8·8%)	414 (9·0%)	394 (8·6%)	221 (9·4%)	211 (7·8%)		193 (8·6%)	183 (9·7%)
75–84	393 (4·3%)	184 (4·0%)	209 (4·6%)	99 (4·2%)	117 (4·3%)		85 (3·8%)	92 (4·9%)
≥85	180 (2·0%)	74 (1·6%)	106 (2·3%)	35 (1·5%)	66 (2·4%)		39 (1·7%)	40 (2·1%)
Level of education attained								
Preschool or no school	1611 (17·5%)	669 (14·5%)	942 (20·5%)	314 (13·4%)	564 (20·9%)		354 (15·7%)	379 (20·0%)
Primary	979 (10·7%)	511 (11·1%)	468 (10·2%)	279 (11·9%)	355 (13·1%)		231 (10·2%)	115 (6·1%)
Secondary or vocational	1543 (16·8%)	1051 (22·9%)	492 (10·7%)	723 (30·9%)	406 (15·0%)		331 (14·7%)	89 (4·7%)
Higher	403 (4·4%)	333 (7·2%)	70 (1·5%)	279 (11·9%)	65 (2·4%)		58 (2·6%)	6 (0·3%)
Do not know or other	154 (1·7%)	43 (0·9%)	111 (2·4%)	6 (0·3%)	47 (1·7%)		37 (1·6%)	64 (3·4%)
Non-formal or Quranic (Islamic)	4498 (49·0%)	1991 (43·3%)	2507 (54·6%)	738 (31·6%)	1263 (46·8%)		1243 (55·1%)	1241 (65·5%)
Ethnicity								
Mandinka	3423 (37·3%)	1573 (34·2%)	1849 (40·3%)	965 (41·3%)	1226 (45·4%)		611 (27·1%)	629 (33·2%)
Wollof	1361 (14·8%)	724 (15·7%)	637 (13·9%)	247 (10·6%)	286 (10·6%)		473 (21·0%)	349 (18·4%)
Jola or Karoninka	1029 (11·2%)	498 (10·8%)	531 (11·6%)	290 (12·4%)	373 (13·8%)		208 (9·2%)	160 (8·4%)
Fula, Tukulor, or Lorobo	2021 (22·0%)	1158 (25·2%)	862 (18·8%)	501 (21·4%)	416 (15·4%)		654 (29·0%)	444 (23·4%)
Sarahuleh	691 (7·5%)	311 (6·8%)	380 (8·3%)	121 (5·2%)	158 (5·9%)		188 (8·3%)	221 (11·7%)
Other	663 (7·2%)	333 (7·2%)	330 (7·2%)	214 (9·1%)	240 (8·9%)		120 (5·3%)	91 (4·8%)
Marital status								
Never married	208 (2·3%)	177 (3·8%)	31 (0·7%)	123 (5·3%)	27 (1·0%)		55 (2·4%)	4 (0·2%)
Married or living together	7817 (85·1%)	4324 (94·0%)	3494 (76·1%)	2147 (91·8%)	2008 (74·4%)		2170 (96·2%)	1488 (78·6%)
Widowed	992 (10·8%)	29 (0·6%)	963 (21·0%)	17 (0·7%)	577 (21·4%)		12 (0·5%)	387 (20·4%)
Divorced or separated	171 (1·9%)	69 (1·5%)	102 (2·2%)	51 (2·2%)	89 (3·3%)		18 (0·8%)	14 (0·7%)
Occupation								
Unemployed	1052 (11·4%)	365 (7·9%)	687 (15·0%)	268 (11·5%)	483 (17·9%)		98 (4·3%)	207 (10·9%)
Manual	4524 (49·2%)	1956 (42·5%)	2569 (56·0%)	442 (18·9%)	1104 (40·9%)		1496 (66·3%)	1456 (76·9%)
Trades	2569 (28·0%)	1492 (32·4%)	1077 (23·5%)	1122 (48·0%)	941 (34·9%)		378 (16·8%)	146 (7·7%)
Professional	650 (7·1%)	563 (12·2%)	87 (1·9%)	380 (16·2%)	76 (2·8%)		185 (8·2%)	12 (0·6%)
Other	163 (1·8%)	146 (3·2%)	17 (0·4%)	72 (3·1%)	12 (0·4%)		74 (3·3%)	5 (0·3%)
Retired or old age	229 (2·5%)	77 (1·7%)	152 (3·3%)	53 (2·3%)	84 (3·1%)		24 (1·1%)	68 (3·6%)
Wealth quintile								
1 (poorest)	870 (9·5%)	481 (10·5%)	389 (8·5%)	44 (1·9%)	29 (1·1%)		431 (19·1%)	356 (18·8%)
2	1418 (15·4%)	794 (17·3%)	624 (13·6%)	153 (6·5%)	126 (4·7%)		634 (28·1%)	492 (26·0%)
3	2238 (24·4%)	1176 (25·6%)	1062 (23·1%)	213 (9·1%)	203 (7·5%)		951 (42·2%)	848 (44·8%)
4	2141 (23·3%)	1039 (22·6%)	1102 (24·0%)	807 (34·5%)	912 (33·8%)		238 (10·6%)	198 (10·5%)
5 (richest)	2520 (27·4%)	1108 (24·1%)	1412 (30·8%)	1122 (48·0%)	1430 (53·0%)		0	0

Data are in mean (SE) or n (%).

**Table 2:** Age and sex-standardised prevalence of hypertension, diabetes, obesity, multimorbidity and related risk factors weighted for cluster size

	All (n=9188)	Men (n=4598)	Women (n=4590)	Urban areas (n=5039)			Rural areas (n=4149)	
				Men (n=2339)	Women (n=2700)		Men (n=2255)	Women (n=1894)
**Hypertension status**								
No	4856 (53·0%)	2535 (55·3%)	2321 (50·7%)	1317 (56·5%)	1374 (51·0%)		1216 (54·0%)	950 (50·3%)
Yes	4311 (47·0%)	2052 (44·7%)	2259 (49·3%)	1014 (43·5%)	1322 (49·0%)		1035 (46·0%)	939 (49·7%)
Missing*	21 (0·2%)	12 (0·3%)	9 (0·2%)	8 (0·4%)	4 (0·2%)		4 (0·2%)	5 (0·3%)
**Diabetes status** †								
No	8611 (93·7%)	4343 (94·5%)	4268 (93·0%)	2180 (93·2%)	2469 (91·4%)		2158 (95·7%)	1802 (95·2%)
Yes	577 (6·3%)	255 (5·6%)	322 (7·0%)	159 (6·8%)	232 (8·6%)		97 (4·3%)	92 (4·8%)
**BMI**								
Underweight	627 (7·2%)	382 (8·7%)	246 (5·6%)	153 (6·9%)	92 (3·6%)		227 (10·6%)	152 (8·5%)
Normal	4904 (56·2%)	2888 (66·0%)	2016 (46·2%)	1413 (63·4%)	1010 (39·1%)		1470 (68·7%)	1003 (56·3%)
Overweight	2151 (24·6%)	933 (21·3%)	1218 (27·9%)	563 (25·3%)	813 (31·5%)		372 (17·4%)	408 (22·9%)
Obese	1050 (12·0%)	170 (3·9%)	880 (20·2%)	100 (4·5%)	665 (25·8%)		71 (3·3%)	220 (12·3)
Missing*	455 (5·0%)	225 (4·9%)	230 (5·0%)	109 (4·7%)	119 (4·4%)		115 (5·1%)	111 (5·9%)
**Number of conditions (hypertension, obesity, or diabetes)**								
None	4117 (47·2%)	2290 (52·5%)	1826 (41·9%)	1177 (53·0%)	1023 (39·7%)		1111 (52·0%)	804 (45·1%)
One	3668 (42·1%)	1835 (42·1%)	1833 (42·1%)	916 (41·2%)	1045 (40·6%)		917 (42·9%)	789 (44·3%)
Two	853 (9·8%)	229 (5·2%)	624 (14·3%)	121 (5·4%)	449 (17·4%)		108 (5·1%)	178 (10·0%)
Three	79 (0·9%)	9·0 (0·2%)	70 (1·6%)	8 (0·4%)	60 (2·3%)		1 (<0·1%)	10 (0·6%)
Missing*	471 (5·1%)	235 (5·1%)	236 (5·1%)	116 (5·0%)	123 (4·6%)		119 (5·3%)	113 (5·9%)
**Alcohol consumption** ‡								
Never	9087 (98·9%)	4523 (98·4%)	4564 (99·4%)	2292 (98·0%)	2693 (99·7%)		2226 (98·7%)	1877 (99·1%)
Ever	101 (1·1%)	75 (1·6%)	26 (0·6%)	47 (2·0%)	8 (0·3%)		29 (1·3%)	17 (0·9%)
**Smoking status** §								
Current smoker	894 (9·7%)	889 (19·3%)	5 (0·1%)	468 (20·0%)	1 (0·1%)		420 (18·6%)	4 (0·2%)
Never smoked	7611 (82·8%)	3028 (65·8%)	4584 (99·9%)	1507 (64·4%)	2699 (99·9%)		1517 (67·3%)	1890 (99·8%)
Previous smoker	682 (7·4%)	682 (14·8%)	1 (<0·1%)	364 (15·6%)	0		318 (14·1%)	0

Data are n (%).

* Missing data are in n (%) of total participants and are not included in the calculation of prevalence estimates.

† Defined as a fasting blood glucose level 7·0 mmol/L or more, random blood glucose of 11·1mmol/L or more, or self-reported history of health personnel diagnosis of diabetes or currently receiving treatment for diabetes.

‡ Self-reported tobacco use.

§ Self-report of any alcohol consumption in the past 12 months.

**Table 3:** Association of risk factors with hypertension and diabetes in the study population, standardised for age and sex

	Hypertension in men			Hypertension in women			Diabetes in men			Diabetes in women	
	Unadjusted	Adjusted*		Unadjusted	Adjusted*		Unadjusted	Adjusted*		Unadjusted	Adjusted*
**Residence**											
Urban	1 (ref)	1 (ref)		1 (ref)	1 (ref)		1 (ref)	1 (ref)		1 (ref)	1 (ref)
Rural	1·11 (0·92–1·33)	1·34 (0·98–1·83)		1·03 (0·91–1·16)	1·15 (0·93–1·41)		0·62 (0·43–0·90)	1·31 (0·74–2·31)		0·54 (0·43–0·68)	0·81 (0·56–1·17)
**Age group, years**											
35–44	1 (ref)	1 (ref)		1 (ref)	1 (ref)		1 (ref)	1 (ref)		1 (ref)	1 (ref)
45–54	1·90 (1·52–2·39)	1·85 (1·46–2·35)		2·34 (2·07–2·64)	2·15 (1·88–2·46)		3·17 (1·81–5·56)	3·21 (1·79–5·75)		1·65 (1·27–2·13)	1·64 (1·24–2·17)
55–64	4·57 (3·64–5·73)	4·04 (3·16–5·15)		3·87 (3·31–4·52)	3·24 (2·72–3·87)		3·62 (2·07–6·32)	3·40 (1·91–6·04)		1·89 (1·45–2·47)	1·74 (1·26–2·40)
65–74	6·78 (5·27–8·71)	5·34 (4·09–6·98)		6·90 (5·47–8·71)	5·05 (3·81–6·69)		3·71 (2·09–6·57)	2·87 (1·49–5·53)		1·94 (1·38–2·71)	1·97 (1·23–3·15)
75–84	7·47 (5·19–10·76)	5·51 (3·59–8·47)		8·06 (5·91–10·98)	4·77 (3·29–6·94)		2·71 (1·31–5·61)	1·51 (0·62–3·72)		1·84 (1·14–2·96)	1·59 (0·81–3·14)
≥85	6·83 (3·79–12·33)	4·57 (2·11–9·91)		9·55 (4·80–19·02)	6·53 (2·65–16·11)		1·89 (0·53–6·69)	2·18 (0·54–8·75)		0·69 (0·16–2·91)	1·35 (0·24–7·65)
**Level of education attained**											
Preschool or no school	1 (ref)	1 (ref)		1 (ref)	1 (ref)		1 (ref)	1 (ref)		1 (ref)	1 (ref)
Primary	0·60 (0·43–0·86)	0·85 (0·58–1·24)		0·62 (0·50–0·77)	0·94 (0·74–1·20)		0·46 (0·22–0·97)	0·50 (0·22–1·16)		1·28 (0·92–1·77)	1·23 (0·84–1·81)
Secondary or vocational	0·64 (0·47–0·85)	0·89 (0·65–1·23)		0·57 (0·46–0·70)	0·87 (0·68–1·12)		0·77 (0·46–1·30)	0·77 (0·41–1·43)		1·15 (0·81–1·62)	1·07 (0·71–1·62)
Higher	0·63 (0·42–0·94)	0·77 (0·49–1·23)		0·61 (0·40–0·95)	0·83 (0·49–1·41)		1·49 (0·73–3·02)	1·32 (0·54–3·24)		0·93 (0·37–2·31)	0·79 (0·26–2·38)
Do not know or other	1·81 (0·92–3·54)	1·05 (0·43–2·58)		1·13 (0·77–1·64)	1·35 (0·84–2·15)		1·23 (0·33–4·67)	1·75 (0·41–7·37)		0·66 (0·31–1·43)	0·77 (0·33–1·81)
Non-formal or Quranic (Islamic)	1·10 (0·84–1·43)	1·05 (0·80–1·38)		0·94 (0·81–1·08)	0·98 (0·83–1·16)		0·72 (0·46–1·12)	0·71 (0·43–1·16)		0·78 (0·60–1·02)	0·85 (0·62–1·16)
**Ethnicity**											
Mandinka	1 (ref)	1 (ref)		1 (ref)	1 (ref)		1 (ref)	1 (ref)		1 (ref)	1 (ref)
Wollof	0·90 (0·69–1·18)	0·82 (0·61–1·10)		0·96 (0·80–1·15)	1·09 (0·90–1·34)		1·01 (0·63–1·63)	1·17 (0·70–1·95)		1·24 (0·92–1·67)	1·33 (0·96–1·85)
Jola or Karoninka	1·03 (0·76–1·41)	0·92 (0·65–1·29)		0·82 (0·69–0·97)	0·76 (0·63–0·92)		0·74 (0·41–1·34)	0·63 (0·31–1·26)		0·74 (0·50·1–10)	0·76 (0·51–1·12)
Fula, Tukulor, or Lorobo	0·92 (0·73–1·17)	0·93 (0·72–1·20)		1·03 (0·88–1·21)	1·22 (1·02–1·46)		0·83 (0·53–1·32)	1·08 (0·65–1·82)		0·80 (0·57–1·12)	0·97 (0·67–1·39)
Sarahuleh	1·58 (1·11–2·26)	1·56 (1·06–2·30)		1·45 (1·19–1·75)	1·59 (1·26–2·00)		0·85 (0·37–1·95)	1·08 (0·45–2·61)		0·84 (0·55–1·27)	0·89 (0·56–1·40)
Other	0·94 (0·62–1·42)	0·81 (0·52–1·28)		1·25 (0·99–1·57)	1·25 (0·95–1·65)		0·68 (0·31–1·48)	0·64 (0·29–1·45)		1·57 (1·05–2·34)	1·50 (1·00–2·27)
**Marital status**											
Never married	1 (ref)	1 (ref)		1 (ref)	1 (ref)		1 (ref)	1 (ref)		1 (ref)	1 (ref)
Married or living together	2·10 (1·25–3·53)	1·14 (0·64–2·03)		1·60 (0·86–2·98)	1·34 (0·69–2·60)		4·51 (0·64–32·02)	2·92 (0·41–20·6)		0·72 (0·25–2·08)	1·21 (0·34–4·29)
Widowed	4·14 (1·20–14·03)	1·31 (0·30–5·76)		5·65 (2·99–10·67)	2·13 (1·09–4·16)		4·38 (0·26–73·54)	4·33 (0·22–85·37)		0·94 (0·33–2·70)	1·33 (0·37–4·77)
divorced or separated	1·99 (0·82–4·84)	1·28 (0·45–3·65)		1·78 (0·89–3·55)	1·43 (0·70–2·91)		6·62 (0·62–70·74)	3·02 (0·24–38·36)		1·54 (0·48–4·94)	1·74 (0·45–6·74)
**Occupation**											
Unemployed	2·32 (1·73–3·11)	1·49 (1·03–2·16)		0·40 (0·33–0·48)	1·35 (1·08–1·67)		1 (ref)	1 (ref)		1 (ref)	1 (ref)
Manual	1 (ref)	1 (ref)		1 (ref)	1 (ref)		0·48 (0·29–0·81)	0·58 (0·31–1·09)		0·63 (0·47–0·82)	0·85 (0·59–1·22)
Trades	0·66 (0·54–0·80)	0·88 (0·69–1·12)		0·37 (0·30–0·45)	0·91 (0·78–1·07)		0·75 (0·45–1·24)	0·91 (0·50–1·66)		1·01 (0·75–1·36)	1·01 (0·69–1·49)
Professional	0·64 (0·48–0·85)	0·98 (0·69–1·39)		0·36 (0·25–0·53)	1·28 (0·82–2·01)		0·85 (0·44–1·65)	0·88 (0·41–1·90)		0·81 (0·39–1·69)	0·87 (0·36–2·15)
Other	0·64 (0·34–1·18)	1·33 (0·88–2·01)		0·87 (0·35–2·15)	1·98 (0·63–6·18)		0·43 (0·13–1·45)	0·63 (0·16–2·56)		4·55 (1·97–10·51)	5·10 (1·94–13·43)
Retired or old age	3·75 (2·15–6·54)	1·17 (0·75–1·84)		2·39 (1·46–3·91)	1·64 (0·98–2·74)		1·46 (0·62–3·44)	1·56 (0·62–3·91)		1·01 (0·53–1·93)	1·09 (0·51–2·36)
**Wealth quintile**											
1 (poorest)	1 (ref)	1 (ref)		1 (ref)	1 (ref)		1 (ref)	1 (ref)		1 (ref)	1 (ref)
2	1·01 (0·73–1·41)	1·13 (0·78–1·65)		0·73 (0·58–0·94)	0·71 (0·54–0·92)		0·82 (0·33–2·04)	0·83 (0·33–2·11)		0·56 (0·35–0·92)	0·51 (0·31–0·84)
3	1·02 (0·74–1·41)	0·94 (0·66–1·35)		0·92 (0·73–1·16)	0·89 (0·69–1·14)		1·82 (0·76–4·35)	1·74 (0·71–4·27)		0·71 (0·48–1·04)	0·63 (0·42–0·93)
4	1·11 (0·81–1·53)	1·30 (0·86–1·97)		0·82 (0·65–1·04)	0·75 (0·56–1·01)		1·53 (0·62–3·79)	1·30 (0·51–3·32)		0·93 (0·65–1·35)	0·61 (0·40–0·93)
5 (richest)	0·97 (0·70–1·33)	1·15 (0·74–1·80)		0·93 (0·74–1·16)	0·89 (0·67–1·20)		2·92 (1·25–6·83)	2·73 (1·02–7·31)		1·61 (1·15–2·25)	0·95 (0·61–1·49)
**BMI**											
Underweight	0·78 (0·57–1·05)	0·63 (0·45–0·88)		0·82 (0·63–1·05)	0·65 (0·49–0·85)		1·01 (0·50–2·02)	1·16 (0·58–2·31)		0·57 (0·30–1·07)	0·56 (0·30–1·06)
Normal	1 (ref)	1 (ref)		1 (ref)	1 (ref)		1 (ref)	1 (ref)		1 (ref)	1 (ref)
Overweight	1·51 (1·21–1·88)	1·64 (1·29–2·09)		1·24 (1·09–1·41)	1·47 (1·28–1·69)		1·87 (1·26–2·77)	1·60 (1·06–2·41)		1·41 (1·08–1·84)	1·26 (0·95–1·66)
Obese	1·88 (1·22–2·89)	1·81 (1·18–2·77)		1·98 (0·72–0·85)	2·58 (2·23–2·98)		2·00 (0·92–4·35)	1·50 (0·62–3·62)		2·09 (1·63–2·67)	1·69 (1·30–2·20)
**Alcohol consumption** †											
Never	1 (ref)	1 (ref)		1 (ref)	1 (ref)		1 (ref)	1 (ref)		1 (ref)	1 (ref)
Ever	1·54 (0·94–2·53)	1·89 (1·02–3·53)		1·52 (0·86–2·66)	0·60 (0·37–0·96)		0·45 (0·07–2·96)	0·58 (0·13–2·60)		1·22 (0·15–9·77)	1·15 (0·15–8·23)
**Smoking status** ‡											
Current smoker	0·57 (0·46–0·71)	0·80 (0·63–1·03)		0·44 (0·83–2·33)	0·31 (0·55·1·70)		0·45 (0·25–0·81)	0·64 (0·35–1·19)		··	··
Never smoked	1 (ref)	1 (ref)		1 (ref)	1 (ref)		1 (ref)	1 (ref)		··	··
Previous smoker	1·08 (0·84–1·39)	1·06 (0·80–1·41)		··	··		1·07 (0·67–1·72)	1·14 (0·69–1·88)		··	··

Data are in OR (95% CI).

* Adjusted for age, ethnicity, education, residence, wealth quintile, occupation, marital status, alcohol consumption, smoking, and BMI.

† Self-report of any alcohol consumption in the past 12 months.

‡ Self-reported smoking.

**Table 4:** Association of risk factors with obesity in the study population, standardised for age and sex

	Men			Women	
	Unadjusted	Adjusted*		Unadjusted	Adjusted*
**Residence**					
Urban	1 (ref)	1 (ref)		1 (ref)	1 (ref)
Rural	0·73 (0·44–1·21)	1·29 (0·66–2·55)		0·40 (0·33–0·49)	0·71 (0·55–0·91)
**Age group, years**					
35–44	1 (ref)	1 (ref)		1 (ref)	1 (ref)
45–54	1·14 (0·62–2·09)	1·16 (0·63–2·17)		1·14 (0·96–1·36)	1·25 (1·04–1·49)
55–64	1·82 (1·03–3·20)	1·82 (0·97–3·40)		1·10 (0·90–1·34)	1·30 (1·04–1·63)
65–74	1·89 (1·02–3·48)	2·01 (0·99–4·08)		0·75 (0·58–0·97	1·00 (0·75–1·35)
75–84	0·58 (1·17–1·96)	0·74 (0·20–2·78)		0·41 (0·25–0·67)	0·56 (0·32–0·99)
≥85	··	··		0·11 (0·02–0·76)	0·15 (0·02–1·00)
**Level of education attained**					
Preschool or no school	1 (ref)	1 (ref)		1 (ref)	1 (ref)
Primary	1·03 (0·39–2·72)	1·05 (0·39–2·80)		2·05 (1·57–2·66)	1·59 (1·22–2·05)
Secondary or vocational	0·92 (0·39–2·13)	0·87 (0·36–2·07)		1·89 (1·48–2·41)	1·35 (1·07–1·72)
Higher	2·19 (0·87–5·04)	1·92 (0·63–5·86)		2·61 (1·72–3·95)	1·69 (1·03–2·77)
Do not know or other	1·24 (0·15–9·97)	1·56 (0·18–13·34)		1·15 (0·71–1·87)	1·21 (0·73–1·99)
Non-formal or Quranic (Islamic)	1·28 (0·65–2·50)	1·33 (0·69–2·59)		0·88 (0·70–1·11)	0·90 (0·73–1·11)
**Ethnicity**					
Mandinka	1 (ref)	1 (ref)		1 (ref)	1 (ref)
Wollof	0·52 (0·26–1·03)	0·48 (0·22–1·04)		0·89 (0·68–1·18)	0·94 (0·74–1·19)
Jola or Karoninka	0·60 (0·26–1·40)	0·57 (0·24–1·33)		1·20 (0·96–1·50)	1·12 (0·90–1·39)
Fula, Tukulor, or Lorobo	0·61 (0·33–1·13)	0·58 (0·13–1·12)		0·73 (0·57–0·92)	0·83 (0·66–1·04)
Sarahuleh	0·40 (0·14–1·16)	0·38 (0·13–1·13)		0·78 (0·57–1·06)	1·02 (0·76–1·37)
Other	0·92 (0·45–1·88)	0·97 (0·44–2·10)		1·45 (1·11–1·90)	1·18 (0·89–1·55)
**Marital status**					
Never married	1 (ref)	1 (ref)		1 (ref)	1 (ref)
Married or living together	1·04 (0·31–3·54)	0·82 (0·23–2·93)		0·60 (0·32–1·14)	1·02 (0·54–1·93)
Widowed	2·98 (0·34–26·5)	4·24 (0·26–69·59)		0·46 (0·24–0·88)	0·93 (0·47–1·82)
Divorced or separated	1·00 (0·1–10·33)	1·08 (0·10–12·18)		1·48 (0·72–3·08)	1·73 (0·83–3·60)
**Occupation**					
Unemployed	1 (ref)	1 (ref)		1 (ref)	1 (ref)
Manual	0·87 (0·39–1·93)	1·17 (0·47–2·87)		0·80 (0·64–1·01)	0·83 (0·65–1·05)
Trades	1·39 (0·65–2·96)	1·99 (0·86–4·59)		2·03 (1·60–2·57)	1·48 (1·16–1·90)
Professional	1·24 (0·51–2·99)	1·31 (0·43–3·99)		1·99 (1·30–3·05)	1·06 (0·63–1·78)
Other	0·72 (0·19–2·76)	1·23 (0·29–5·22)		1·51 (0·56–4·07)	1·23 (0·44–3·43)
Retired or old age	··	··		0·55 (0·31–0·96)	0·93 (0·52–1·64)
**Wealth quintile**					
1 (poorest)	1 (ref)	1 (ref)		1 (ref)	1 (ref)
2	0·92 (0·35–2·78)	1·00 (0·37–2·73)		0·93 (0·64–1·35)	0·91 (0·62–1·34)
3	1·69 (0·71–4·03)	1·69 (0·68–4·16)		0·94 (0·65–1·35)	0·90 (0·62–1·29)
4	1·75 (0·67–4·55)	1·85 (0·69–5·00)		1·79 (1·26–3·42)	1·11 (0·76–1·60)
5 (richest)	2·08 (0·82–5·29)	1·94 (0·68–5·50)		2·44 (1·73–3·43)	1·30 (0·88–1·92)
**Alcohol consumption** †					
Never	1 (ref)	1 (ref)		1 (ref)	1 (ref)
Ever	1·15 (0·32–4·11)	1·00 (0·26–12·18)		0·96 (0·54–1·72)	1·14 (0·64–2·00)
**Smoking status** ‡					
Current smoker	0·39 (0·19–0·80)	0·39 (0·19–0·82)		··	··
Never smoked	1 (ref)	1 (ref)		··	··
Previous smoker	0·98 (0·56–1·71)	0·98 (0·26–3·78)		··	··

Data are in OR (95% CI).

* Adjusted for age, ethnicity, education, residence, wealth quintile, occupation, marital status, alcohol consumption, smoking, and BMI.

† Self-report of any alcohol consumption in the past 12 months.

‡ Self-reported smoking.

## Data Availability

Survey content is available upon request. For any data requests, please contact Islay Mactaggart (islay.mactaggart@lshtm.ac.uk). Additional documents (eg, study questionnaire and informed consent sheet) are available are at https://doi.org/10.17605/OSF.IO/EKCDT.

## Research in context

### Evidence before this study

We did a literature search to identify cross-sectional population surveys in sub-Saharan Africa on the burden of hypertension, diabetes, obesity, and multimorbidity. We searched PubMed for articles using the search string “Africa South of the Sahara”, “hypertension”, “glucose metabolism disorders”, “body mass index”, “comorbidity”, and “epidemiological studies”, published between Jan 1, 1990, and July 1, 2022, without language restrictions. There have been several such published studies across sub-Saharan Africa. Prevalence of hypertension, diabetes, obesity, and multimorbidity, respectively, varied between studies, and there were variations in prevalence between sex and residence. There were few studies focusing on middle-aged and older adults. We found only two nationwide surveys including both sexes in The Gambia that were done in 1996 and 2010, including age ranges 15 years or more and 25–64 years, respectively. Besides none of these being recent studies and not focusing on middle-aged and older adults who are disproportionately affected by these chronic conditions, they also did not investigate the clustering of comorbidities. Our research set out to evaluate the prevalence of hypertension, diabetes, obesity, and multimorbidity in middle-aged and older adults in The Gambia.

### Added value of this study

Our study provides urgently needed recent data on the prevalence of non-communicable diseases (NCDs) in adults aged 35 years or older in The Gambia. Our findings show a high prevalence of hypertension, diabetes, obesity, and multimorbidity in middle-aged and older adults. Hypertension was particularly high, higher in women than men, and similar in urban and rural areas. The prevalence of diabetes was similar in men and women overall, but women from urban areas were more affected than their rural counterparts, with men in both settings similarly affected. Obesity was five-fold higher in women than in men. Multimorbidity also disproportionately affected women, especially in urban areas. Smoking was almost exclusively in men whereas alcohol consumption was very low in both sexes.

### Implications of all the available evidence

The high burden of NCDs and related risk factors is alarming for a country that is already struggling to control infectious diseases, as well as high maternal and under-five mortality rates. This high burden, coupled with overall increasing life-expectancy and rapid unplanned urbanisation, further highlights the need for urgent concerted multisectoral approach to NCDs in The Gambia. The country launched a multisectoral strategy and costed action plan in 2022 to tackle NCDs and this study will help to inform the delivery of this plan and similar local initiatives.
