# Supplementary material for: Prevalence of hypertension, diabetes, obesity, multimorbidity, and related risk factors among adult Gambians: a cross-sectional nationwide study
Source: Lancet Glob Health. Author manuscript; Available in PMC 2025 Oct 18. (PMC7618049; doi:10.1016/S2214-109X(23)00508-9)
Supplement: Supplementary appendix 1 [file EMS208202-supplement-Supplementary_appendix_1.pdf]

THE LANCET
Global Health

Supplementary appendix 1

This appendix formed part of the original submission and has been peer reviewed. We post it as supplied by the authors.

Supplement to: Jobe M, Mactaggart I, Bell S, et al. Prevalence of hypertension, diabetes, obesity, multimorbidity, and related risk factors among adult Gambians: a cross-sectional nationwide study. *Lancet Glob Health* 2024; **12:** e55–65.

SUPPLEMENTAL APPENDIX

Accompanying the manuscript

Prevalence of hypertension, diabetes, obesity, multimorbidity and related risk factors among adult Gambians: a nationwide survey

Modou Jobe FWACP[1,*], Islay Mactaggart PhD[2], Suzannah Bell MBChB[3], Min J Kim MPH[2], Abba Hydara M Med Opthalmol[4], Covadonga Bascaran MSc[2], Modou Njai MSc[5], Omar Badjie MSc[5], Pablo Perel PhD[6], Andrew M Prentice PhD[1], Matthew J Burton PhD[2,7]

[1] Medical Research Council Unit The Gambia at London School of Hygiene and Tropical Medicine, Fajara, The Gambia
[2] International Centre for Eye Health, London School of Hygiene & Tropical Medicine.
[3] Moorfields Eye Hospital NHS Foundation Trust, London, UK
[4] Sheikh Zayed Regional Eye Care Centre, Banjul, Gambia
[5] Directorate of Health Promotion & Education, Ministry of Health, The Gambia
[6] Department of Non-communicable Disease Epidemiology, London School of Hygiene & Tropical Medicine, London, UK
[7] National Institute for Health Research Biomedical Research Centre for Ophthalmology, Moorfields Eye Hospital NHS Foundation Trust, London, UK

Table of Content

Supplementary Figure 1: Conceptual Framework of risk factors for outcomes of diabetes and hypertension 2

Supplementary Figure 2: Unadjusted odds of hypertension and diabetes in men and women by I) body mass index category (normal weight as reference group) and II) Wealth Quintile (quintile 1 as reference group) III) Odds ratio of hypertension (A) and diabetes (B) in men compared to women by residence 3

Supplementary Table 1: Sensitivity analysis of socio-economic position and vision impairment before and after imputation 4

Supplementary Table 2: Unadjusted and non-weighted socio-demographic characteristics of study participants 5

Supplementary Table 3: Age and sex-standardised prevalence (95% CI) of hypertension by selected socio-demographic characteristics and risk factors weighted for cluster size 6

Supplementary Table 4: Age and sex-standardised prevalence (95% CI) of diabetes by selected socio-demographic characteristics weighted for cluster size 8

Supplementary Table 5: Age and sex-standardised prevalence (95% CI) of obesity by selected socio-demographic characteristics and risk factors weighted for cluster size 10

Supplementary Table 6: Age and sex-standardised prevalence (95% CI) of multimorbidity by selected socio-demographic characteristics and risk factors weighted for cluster size 12

Supplementary Table 7: Association of risk factors with hypertension and diabetes in the study population, adjusted for non-modifiable and contextual factors* 14

Supplementary Table 8: Association of risk factors with obesity in the study population, adjusted for non-modifiable and contextual factors* 16

Supplementary Figure 1: Conceptual Framework of risk factors for outcomes of diabetes and hypertension

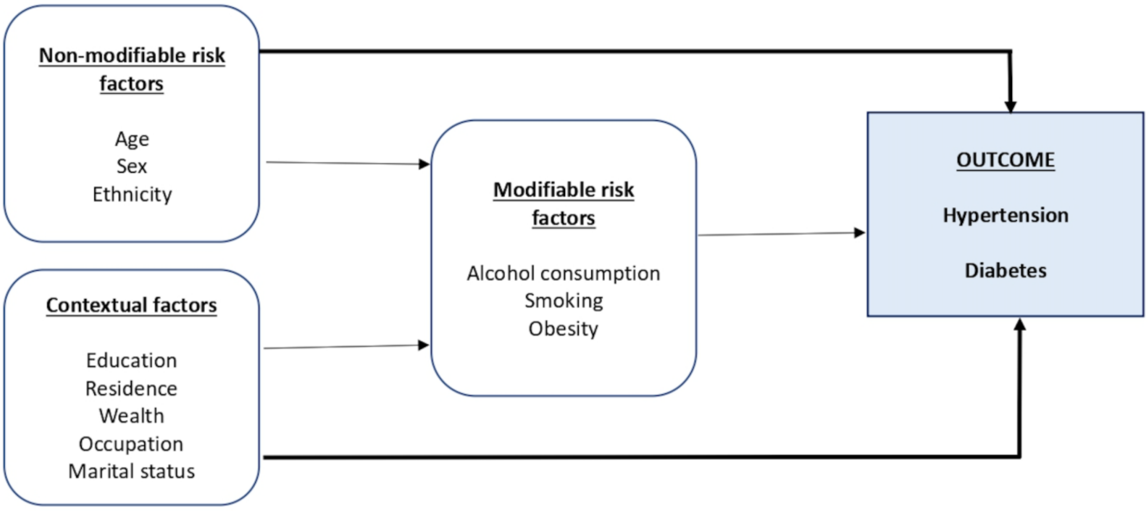

Supplementary Figure 2: Unadjusted odds of hypertension and diabetes in men and women by I) body mass index category (normal weight as reference group) and II) Wealth Quintile (quintile 1 as reference group) III) Odds ratio of hypertension (A) and diabetes (B) in men compared to women by residence

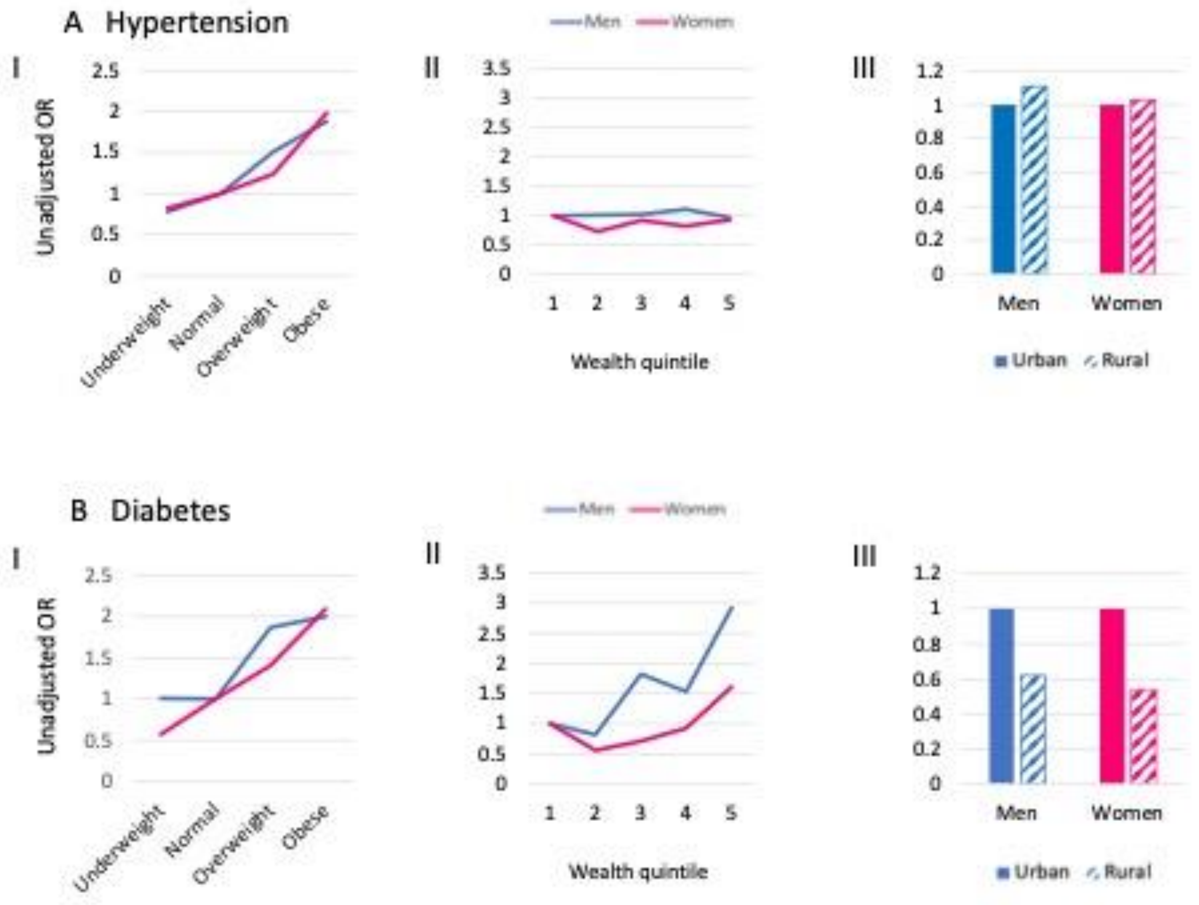

Supplementary Table 1: Sensitivity analysis of socio-economic position and vision impairment before and after imputation

		All surveyed (9788)		WITHOUT IMPUTATION				AFTER IMPUTATION			
				Dataset without imputation		Excluded		Final dataset*		Excluded	
All		9,309		8,790		519		9,188		121	
Presenting vision (corrected)											
	1: can see 6/12	7,977	85.7%	7,530	85.7%	447	86.1%	7,861	89.4%	116	22.4%
	2: cannot see 6/12, but can see 6/18	326	3.5%	316	3.6%	10	1.9%	326	3.7%	0	0.0%
	3: cannot see 6/18, but can see 6/60	728	7.8%	687	7.8%	41	7.9%	726	8.3%	2	0.4%
	4: cannot see 6/60, but can see 3/60	173	1.9%	164	1.9%	9	1.7%	171	1.9%	2	0.4%
	5: cannot see 3/60, but can see 1/60	105	1.1%	93	1.1%	12	2.3%	104	1.2%	1	0.2%
Age categories (years)											
	35-44	4,154	44.6%	3,930	44.7%	224	43.2%	4,102	46.7%	52	10.0%
	45-54	2,095	22.5%	1,978	22.5%	117	22.5%	2,061	23.4%	34	6.6%
	55-64	1,462	15.7%	1,389	15.8%	73	14.1%	1,444	16.4%	18	3.5%
	65-74	1,031	11.1%	956	10.9%	75	14.5%	1,018	11.6%	13	2.5%
	75-84	444	4.8%	422	4.8%	22	4.2%	441	5.0%	3	0.6%
	85+	123	1.3%	115	1.3%	8	1.5%	122	1.4%	1	0.2%
Sex											
	Men	2,753	29.6%	2,593	29.5%	160	30.8%	2,710	30.8%	43	8.3%
	Women	6,556	70.4%	6,197	70.5%	359	69.2%	6,478	73.7%	78	15.0%

*final dataset obtained after exclusion of 479 participants with incomplete/missing/inaccurate core variables. Follow-up data collection was then conducted after which we excluded another 121 that have no socio-economic status variables.

Supplementary Table 2: Unadjusted and non-weighted socio-demographic characteristics of study participants

	Total			Urban (N=5039)		Rural (N=4149)	
	**All (N=9188)**	**M (N=2710)**	**W (N=6478)**	**M (N=1336)**	**W (N=3703)**	**M (N=1374)**	**W (N=2775)**
**Age**							
Mean (SD)	49.6 (13.4)	54.1 (14.0)	47.7 (12.7)	54.6 (14.0)	47.6 (12.6)	53.6 (14.0)	47.9 (12.8)
Age categories							
35-44	4012 (44.7%)	827 (30.5%)	3275 (50.6%)	400 (29.9%)	1867 (50.4%)	427 (31.1%)	1408 (50.7%)
45-54	2061 (22.4%)	615 (22.7%)	1446 (22.3%)	277 (20.7%)	849 (22.9%)	338 (24.6%)	597 (21.5%)
55-64	1444 (15.7%)	557 (20.6%)	887 (13.7%)	294 (22.0%)	523 (14.1%)	263 (19.2%)	364 (13.1%
65-74	1018 (11.1%)	462 (17.1%)	556 (8.6%)	240 (18.0%)	290 (7.8%)	222 (16.2%)	266 (9.6%)
75-84	441 (4.8%)	185 (6.8%)	256 (4.0%)	96 (7.2%)	139 (3.8%)	89 (6.5%)	117 (4.2%)
85+	122 (1.3%)	64 (2.4%)	58 (0.9)	29 (2.2%)	35 (1.0%)	35 (2.6%)	23 (0.8%)
**Level of education attained**							
Pre-school/no school	1651 (18.0%)	401 (14.8%)	1250 (19.3%)	186 (13.9%)	711 (19.2%)	214 (15.6%)	539 (19.4%)
Primary	985 (10.7%)	265 (9.8%)	720 (11.1%)	144 (10.8%)	521 (14.1%)	121 (8.8%)	199 (7.2%)
Secondary/ vocational	1316 (14.3%)	547 (20.2%)	769 (11.9%)	371 (27.8%)	616 (16.6%)	176 (12.8%)	153 (5.5%)
Higher	286 (3.1%)	175 (6.5%)	111 (1.7%)	145 (10.9%)	99 (2.7%)	30 (3.2%)	12 (0.4%)
Don't know/other	189 (2.1%)	30 (1.1%)	159 (2.5%)	5 (0.4%)	66 (1.8%)	25 (1.8%)	93 (3.4%)
non-formal/Quranic	4761 (51.8%)	1291 (47.7%)	3469 (53.6%)	485 (36.3%)	1690 (45.6%)	807 (58.7%)	1779 (64.1%)
**Ethnicity**							
Mandinka	3564 (38.8%)	945 (34.9%)	2619 (40.3%)	559 (41.8%)	1693 (45.7%)	386 (28.1%)	926 (33.4%)
Wollof	1365 (14.9%)	439 (16.2%)	926 (14.3%)	149 (11.2%)	398 (10.8%)	290 (21.1%)	528 (19.0%)
Jola/Karoninka	1079 (11.7%)	312 (11.5%)	767 (11.8%)	181 (13.6%)	524 (14.2%)	131 (9.5%)	243 (8.8%)
Fula/Tukulor/Lorobo	1847 (20.1%)	638 (23.5%)	1209 (18.7%)	256 (19.2%)	554 (15.0%)	382 (27.8%)	655 (23.6%)
Sarahuleh	677 (7.4%)	175 (6.5%)	502 (7.8%)	69 (5.2%)	215 (5.8%)	106 (7.7%)	287 (10.3%)
Others	656 (7.1%)	201 (7.4%)	455 (7.0%)	122 (9.1%)	319 (8.6%)	79 (5.8%)	136 (4.9%)
**Marital status**							
never married	132 (1.4%)	81 (3.0%)	51 (0.8%)	55 (4.1%)	42 (1.1%)	26 (1.9%)	9 (0.3%)
married/living together	7641 (83.2%)	2573 (94.9%)	5068 (78.2%)	1244 (93.1%)	2835 (76.6%)	1329 (96.7%)	2233 (80.5%)
widowed	1229 (13.4%)	20 (0.7%)	1209 (18.7%)	12 (0.9%)	699 (18.9%)	8 (0.6%)	510 (18.4%)
divorced/separated	186 (2.0%)	36 (1.3%)	150 (2.3%)	25 (1.9%)	127 (3.4%)	11 (0.8%)	23 (0.8%)
**Occupation**							
Unemployed	1178 (12.8%)	302 (11.1%)	876 (13.5%)	210 (15.7%)	605 (16.3%)	92 (6.7%)	271 (9.8%)
manual	4931 (53.7%)	1193 (44.0%)	3738 (57.7%)	274 (20.5%)	1563 (42.2%)	919 (66.9%)	2175 (78.4%)
Trades	2304 (25.1%)	768 (28.3%)	1536 (23.7%)	571 (42.7%)	1319 (35.6%)	197 (14.3%)	217 (7.8%)
professional	427 (4.7%)	292 (10.8%)	135 (2.1%)	192 (14.4%)	113 (3.1%)	100 (7.3%)	22 (0.8%)
other	108 (1.2%)	83 (3.1%)	25 (0.4%)	41 (3.1%)	17 (0.5%)	42 (3.1%)	8 (0.3%)
retired/old age	240 (2.6%)	72 (2.7%)	168 (2.6%)	48 (3.6)	86 (2.3%)	24 (1.8%)	82 (3.0%)
**Wealth quintile**							
1 (poorest)	853 (9.3%)	289 (10.7%)	564 (8.7%)	27 (2.0%)	34 (0.9%)	262 (19.1%)	530 (19.1%)
2	1313 (14.3%)	437 (16.1%)	876 (13.%)	75 (5.6%)	165 (4.5%)	362 (26.4%)	711 (25.6%)
3	2251 (24.5%)	729 (26.9%)	1522 (23.5%)	118 (8.8%)	270 (7.3%)	611 (44.5%)	1252 (45.1%)
4	2121 (23.1%)	597 (22.0%)	1524 (23.5%)	458 (34.3%)	1242 (33.5%)	139 (10.1%)	282 (10.2%)
5 (richest)	2650 (28.8%)	658 (24.3%)	1992 (30.8%)	658 (49.3%)	1992 (53.8%)	0	0

Supplementary Table 3: Age and sex-standardised prevalence (95% CI) of hypertension by selected socio-demographic characteristics and risk factors weighted for cluster size

	Total			Urban		Rural	
	**All**	**M**	**W**	**M**	**W**	**M**	**W**
**All**	47.0 (45.6-48.5)	44.7 (42.4-47.0)	49.3 (47.8-50.8)	43.5 (40.4-46.6)	49.0 (47.2-50.9)	46.0 (42.6-49.3)	49.7 (47.2-52.2)
**Age categories (years)**							
35-44	30.5 (28.7-32.4)	28.5 (25.3-31.7)	32.4 (30.6-34.2)	27.3 (22.8-31.7)	31.6 (29.4-33.8)	29.8 (25.2-34.4)	33.5 (30.5-36.5)
45-54	47.9 (45.3-50.5)	43.2 (38.8-47.5)	52.8 (50.0-55.6)	40.4 (34.7-46.1)	53.9 (50.3-57.4)	45.7 (39.3-52.2)	51.2 (46.9-55.6)
55-64	64.8 (62.2-67.3)	64.6 (60.6-68.6)	65.0 (60.6-68.6)	65.4 (59.8-71.0)	66.0 (62.1-69.9)	63.7 (58.0-69.3)	63.4 (58.3-68.5)
65-74	74.9 (72.0-77.7)	73.0 (69.0-77.0)	76.8 (72.8-80.8)	71.1 (65.6-76.6)	74.2 (68.6-79.9)	75.1 (69.2-81.0)	79.7 (74.1-85.2)
75-84	77.3 (73.2-81.4)	74.9 (68.4-81.4)	79.4 (74.4-84.4)	65.5 (55.8-75.2)	79.2 (72.5-85.9)	85.3 (77.9-92.8)	79.7 (72.1-87.3)
85+	78.3 (70.9-85.8)	73.2 (62.1-84.3)	82.1 (72.0-92.2)	73.9 (57.6-90.3)	77.2 (63.3-91.1)	72.3 (57.4-87.6)	90.6 (78.0-1.03)
**Level of education attained**							
Pre-school/no school	51.0 (47.8-54.3)	48.3 (42.6-54.0)	53.0 (50.1-55.9)	50.5 (42.4-58.5)	55.1 (51.5-58.8)	46.4 (38.2-54.6)	49.9 (45.2-54.5)
Primary	38.5 (34.5-42.5)	36.1 (29.7-42.5)	41.2 (37.0-45.4)	34.7 (26.1-42.2)	42.0 (37.0-46.9)	37.7 (28.0-47.4)	39.0 (31.6-46.4)
Secondary/ vocational	37.8 (34.5-41.0)	37.2 (32.9-41.6)	38.9 (34.9-42.9)	36.4 (31.2-41.7)	39.0 (34.5-43.4)	38.9 (31.2-46.6)	38.8 (29.9-47.8)
Higher	37.6 (31.0-44.1)	36.9 (29.3-44.5)	40.9 (30.8-51.0)	38.0 (29.7-46.3)	40.8 (30.0-51.5)	31.7 (13.4-50.0)	41.8 (13.5-70.2)
Don't know/other	57.9 (49.8-66.0)	62.8 (48.1-77.5)	56.0 (47.1-64.8)	82.7 (51.1-1.14)	56.6 (43.1-70.2)	59.9 (44.5-75.4)	55.5 (43.9-67.1)
non-formal/Quranic	51.1 (49.0-53.1)	50.6 (47.2-54.0)	51.4 (49.3-53.5)	52.5 (47.6-57.5)	51.7 (49.1-54.3)	49.5 (45.0-54.0)	51.2 (47.9-54.4)
**Ethnicity**							
Mandinka	47.0 (44.9-49.0)	44.9 (41.3-48.5)	48.7 (46.5-50.9)	41.5 (36.8-46.1)	47.9 (45.4-50.4)	50.1 (44.6-55.5)	50.4 (46.1-54.6)
Wollof	44.9 (41.4-48.4)	42.4 (36.9-47.9)	47.8 (43.6-51.9)	44.4 (35.2-53.6)	50.6 (45.4-55.7)	41.3 (34.5-48.1)	45.5 (39.3-51.7)
Jola/Karoninka	44.7 (40.8-48.6)	45.7 (39.0-52.4)	43.7 (39.9-47.6)	49.2 (41.5-56.9)	41.6 (37.4-45.8)	40.9 (29.6-52.3)	48.6 (40.5-56.7)
Fula/Tukulor/Lorobo	45.7 (42.5-49.0)	42.9 (38.2-47.6)	49.5 (46.2-52.9)	43.0 (35.2-50.8)	51.7 (46.5-57.0)	42.9 (37.1-48.6)	47.5 (43.3-51.8)
Sarahuleh	57.2 (52.5-61.8)	56.3 (48.2-64.3)	57.9 (53.8-62.0)	47.8 (36.8-58.7)	55.7 (50.0-61.4)	61.6 (50.7-72.6)	59.4 (53.6-65.3)
Others	48.7 (42.9-54.6)	43.3 (33.9-52.6)	54.3 (49.1-59.4)	42.6 (32.2-53.0)	55.7 (50.0-61.7)	44.4 (26.2-62.7)	50.6 (41.0-60.1)
**Marital status**							
never married	28.9 (19.7-38.0)	28.3 (18.0-38.6)	32.2 (18.7-45.7)	32.0 (18.9-45.0)	31.1 (16.5-45.7)	20.3 (4.8-35.7)	38.6 (3.4-73.8)
married/living together	44.3 (42.7-45.9)	45.3 (42.9-47.7)	43.1 (41.4-44.9)	44.0 (40.7-47.2)	43.2 (41.0-45.4)	46.6 (43.2-50.0)	43.0 (40.2-45.8)
widowed	72.5 (69.9-75.2)	62.0 (35.2-88.8)	72.9 (70.3-75.5)	62.1 (33.1-91.2)	70.6 (67.3-74.0)	61.9 (11.4-112.3)	76.1 (72.1-80.1)
divorced/separated	45.1 (37.2-52.9)	44.0 (26.6-61.4)	45.8 (38.6-53.0)	45.7 (24.9-66.6)	46.7 (39.1-54.2)	39.2 (7.8-70.6)	40.3 (19.0-61.6)
**Occupation**							
unemployed	67.6 (64.5-70.3)	67.8 (3.1-73.9)	67.5 (63.8-71.2)	67.2 (59.7-74.7)	66.6 (62.1-71.0)	69.5 (59.8-79.2)	69.7 (63.0-76.4)
manual	46.2 (44.2-48.2)	47.6 (44.3-51.0)	45.1 (43.1-47.2)	46.8 (40.4-53.2)	44.7 (41.8-47.6)	47.9 (44.0-51.8)	45.5 (42.6-48.4)
trades	39.8 (37.4-42.3)	37.4 (33.6-41.2)	43.2 (40.4-46.1)	36.7 (32.1-41.2)	42.6 (39.4-45.7)	39.5 (32.8-46.2)	47.4 (40.6-54.3)
professional	37.6 (32.5-42.7)	36.7 (31.0-42.4)	43.0 (34.4-51.5)	37.6 (30.6-44.6)	41.8 (32.7-51.0)	35.0 (25.2-44.7)	50.1 (25.7-74.5)
other	39.6 (26.4-52.9)	36.6 (22.8-50.5)	64.5 (44.1-84.9)	47.8 (31.1-64.4)	67.3 (42.9-91.7)	26.1 (8.7-43.4)	57.8 (23.3-92.4)
retired/old age	81.3 (76.1-86.4)	77.3 (67.8-86.8)	83.3 (76.8-89.7)	77.0 (65.6-88.5)	82.2 (72.0-92.3)	78.0 (61.0-95.0)	84.6 (77.3-91.8)
**BMI**							
Underweight	36.9 (32.0-41.8)	35.5 (28.9-42.2)	39.0 (33.1-44.9)	26.4 (17.0-35.8)	42.7 (33.1-52.3)	41.5 (32.7-50.4)	36.8 (29.4-44.2)
Normal	42.5 (40.6-44.4)	41.5 (38.8-44.3)	43.9 (41.8-46.0)	39.8 (36.2-43.5)	41.7 (38.7-44.7)	43.1 (39.1-47.1)	46.0 (43.2-48.9)
Overweight	50.3 (47.6-53.0)	51.7 (46.7-56.7)	49.3 (46.6-51.9)	50.4 (43.4-57.4)	47.5 (44.3-50.6)	53.6 (46.9-60.3)	52.7 (47.9-57.6)
Obese	60.0 (57.2-62.9)	57.1 (47.0-67.3)	60.6 (57.7-63.5)	53.4 (40.5-66.4)	59.5 (56.2-62.8)	62.2 (46.4-78.0)	63.8 (58.1-69.6)
**Family history of hypertension[a]**							
No	40.5 (38.5-42.6)	40.6 (37.6-43.6)	40.4 (38.1-42.7)	39.7 (35.7-43.8)	39.3 (36.7-41.9)	41.7 (37.2-46.2)	42.1 (38.1-46.0)
Yes	52.0 (49.9-54.1)	46.8 (43.1-50.4)	56.5 (54.5-58.5)	45.2 (40.0-50.5)	57.1 (54.5-59.6)	48.0 (43.0-52.9)	55.7 (52.5-59.0)
Don't know	53.2 (49.9-56.5)	53.8 (48.5-59.0)	52.6 (49.1-56.2)	55.6 (48.7-62.5)	53.3 (48.5-58.1)	52.3 (44.7-60.0)	51.9 (46.7-57.0)
**Alcohol consumption[b]**							
Never	46.9 (45.5-48.3)	44.6 (42.3-46.8)	49.3 (47.8-50.8)	43.1 (40.1-46.2)	49.0 (47.1-50.9)	46.0 (42.6-49.4)	49.7 (47.1-52.2)

Ever	56.3 (46.9-65.8)	55.3 (43.1-67.4)	59.5 (45.8-73.1)	61.5 (45.0-78.0)	64.0 (39.1-88.9)	45.4 (26.8-64.0)	57.5 (41.6-73.4)
**Smoking status[c]**							
Current smoker	33.7 (29.1-38.3)	33.7 (29.1-38.3)	29.9 (-5.2-65.0)	33.6 (26.7-40.5)	0.00	33.8 (27.7-39.8)	39.5 (-4.4 - 83.5)
Never smoked	48.4 (46.9-49.9)	47.0 (44.4-49.7)	49.3 (47.8-50.9)	44.9 (41.4-48.5)	49.1 (47.2-50.9)	49.0 (45.2-52.9)	49.7 (47.2-52.2)
Previous smoker	49.0 (43.1-54.9)	48.9 (43.0-54.8)	0.00	50.3 (41.4-59.2)	0.00	47.4 (40.0-54.8)	0.00
**Wealth quintile**							
1 (poorest)	47.9 (43.2-52.6)	44.2 (37.5-50.8)	52.6 (47.6-57.7)	44.0 (22.5-65.5)	43.1 (22.1-64.2)	44.2 (37.2-51.1)	53.4 (48.2-58.5)
2	44.7 (41.4-47.9)	44.5 (39.2-49.7)	44.9 (41.0-48.8)	39.0 (26.9-51.1)	43.7 (35.8-51.7)	45.7 (39.9-51.5)	45.2 (40.8-49.7)
3	47.5 (44.6-50.4)	44.7 (40.4-49.0)	50.6 (47.5-53.6)	40.6 (31.9-49.3)	50.7 (44.6-56.9)	45.6 (40.7-50.5)	50.5 (47.0-54.0)
4	47.2 (44.5-50.0)	46.8 (42.4-51.2)	47.7 (44.8-50.6)	45.4 (40.3-50.4)	46.9 (43.7-50.2)	51.4 (42.6-60.1)	51.1 (44.2-58.0)
5 (richest)	47.5 (45.2-49.8)	43.3 (39.2-47.4)	50.7 (48.3-53.2)	43.3 (39.2-47.4)	50.7 (48.3-53.2)	0.00	0.00
**Diabetes status[d]**							
No	45.7 (44.2-47.3)	43.7 (41.3-46.1)	47.8 (46.3-49.4)	42.3 (39.0-45.5)	47.0 (45.1-48.9)	45.1 (41.7-48.5)	49.0 (46.4-51.5)
Yes	66.1 (61.7-70.4)	62.2 (54.7-69.8)	69.1 (64.5-73.8)	60.1 (50.0-70.2)	71.0 (65.5-76.5)	65.6 (54.5-76.6)	64.5 (55.9-73.1)

[a] self-report; [b] self-report of any alcohol consumption in the past 12 months; [c] self-reported tobacco use; [d] defined as a fasting blood glucose level ≥7mmol/L or random blood glucose of ≥11.1mmol/L and/or self-reported history of health personnel diagnosis of diabetes and/or currently receiving treatment for diabetes

Abbreviations: M=men; W=women

Supplementary Table 4: Age and sex-standardised prevalence (95% CI) of diabetes by selected socio-demographic characteristics weighted for cluster size

	Total			Urban		Rural	
	All	M	W	M	W	M	W
**All**	6.3 (5.7-6.9)	5.6 (4.6-6.5)	7.0 (6.3-7.7)	6.8 (5.3-8.2)	8.6 (7.6-9.6)	4.3 (3.1-5.5)	4.8 (4.0-5.7)
**Age categories**							
35-44	3.8 (3.1-4.6)	2.5 (1.4-3.6)	5.1 (4.3-5.9)	2.2 (0.8-3.6)	5.9 (4.8-7.0)	2.8 (1.0-4.5)	4.1 (3.0-5.3)
45-54	7.8 (6.5-9.2)	7.4 (5.3-9.7)	8.2 (6.7-9.7)	10.5 (6.7-14.4)	9.8 (7.7-12.0)	4.8 (2.6-7.0)	5.7 (3.8-7.6)
55-64	8.9 (7.2-10.5)	8.5 (6.0-11.0)	9.3 (7.3-11.2)	10.7 (6.9-14.5)	11.9 (9.1-14.7)	5.9 (2.9-8.9)	5.4 (3.1-7.6)
65-74	9.1 (7.2-10.9)	8.7 (6.1-11.3)	9.5 (6.9-12.1)	9.8 (5.9-13.6)	13.0 (8.8-17.2)	7.5 (3.9-11.0)	5.5 (2.8-8.2)
75-84	7.8 (5.1-10.5)	6.5 (2.9-10.1)	9.0 (5.4-12.7)	7.2 (2.0-12.5)	12.6 (6.8-18.5)	5.6 (0.8-10.5)	4.5 (1.0-8.0)
85+	4.0 (0.4-7.6)	4.6 (-0.5-9.8)	3.6 (-1.3-8.6)	6.7 (-2.4- 15.8)	2.8 (-2.7-8.3)	2.8 (-2.6-8.2)	4.9 (-4.5- 14.2)
**Level of education attained**							
Pre-school/no school	7.3 (6.0-8.7)	6.9 (4.5-9.2)	7.6 (6.1-9.2)	9.3 (5.8-12.8)	9.2 (7.1-11.4)	4.8 (1.7-7.9)	5.3 (3.3-7.3)
Primary	6.3 (4.7-7.8)	3.3 (1.2-5.4)	9.5 (7.1-11.9)	3.2 (0.5-5.8)	10.9 (7.9-13.9)	3.4 (0.1-6.7)	5.3 (2.0-8.6)
Secondary/ vocational	6.4 (4.9-7.9)	5.4 (3.4-7.3)	8.7 (6.5-10.8)	6.4 (3.9-9.0)	9.7 (7.2-12.2)	3.1 (0.5-5.8)	4.1 (1.0-7.4)
Higher	9.4 (4.9-13.9)	9.9 (4.6-15.2)	7.1 (1.3-12.9)	10.5 (4.4-16.6)	7.8 (1.4-14.1)	7.1 (-2.1-16.4)	0
Don't know/other	6.1 (2.2-10.0)	8.3 (-1.3-18.0)	5.2 (1.6-8.8)	0	9.6 (1.9-17.2)	9.5 (-1.7-20.8)	2.1 (-0.8-4.9)
non-formal/Quranic	5.6 (4.8-6.4)	5.0 (3.8-6.2)	6.1 (5.2-6.9)	6.1 (3.9-8.3)	7.3 (6.0-8.5)	4.4 (3.0-5.8)	4.9 (3.8-6.0)
**Ethnicity**							
Mandinka	6.7 (5.7-7.6)	6.1 (4.6-7.7)	7.1 (6.0-8.1)	7.0 (4.9-9.1)	8.1 (6.7-9.5)	4.9 (2.5-7.3)	5.2 (3.8-6.6)
Wollof	7.3 (5.8-8.9)	6.2 (3.9-8.5)	8.6 (6.6-10.6)	5.4 (2.3-8.6)	11.2 (7.7-14.7)	6.6 (3.5-9.8)	6.6 (4.4-8.8)
Jola/Karoninka	5.0 (3.4-6.6)	4.6 (2.3-7.0)	5.4 (3.5-7.2)	6.6 (2.9-10.4)	6.5 (4.0-9.0)	1.9 (-0.07-3.9)	2.7 (1.0-4.4)
Fula/Tukulor/Lorobo	5.4 (4.1-6.7)	5.2 (3.4-7.0)	5.7 (4.1-7.4)	7.3 (4.0-10.6)	7.3 (4.9-9.6)	3.6 (1.7-5.5)	4.4 (2.1-6.6)
Sarahuleh	5.7 (3.7-7.7)	5.3 (1.4-9.1)	6.0 (3.9-8.1)	6.9 (-0.7-14.6)	8.3 (4.2-12.4)	4.2 (-0.05-8.5)	4.4 (2.3-6.5)
Others	7.4 (5.1-9.8)	4.3 (1.3-7.2)	10.7 (7.2-14.1)	6.4 (2.0-10.8)	13.7 (9.4-18.0)	0.6 (-0.6-1.8)	2.9 (-0.04-5.8)
**Marital status**							
never married	2.4 (-0.07-4.9)	1.3 (-1.2-3.9)	8.7 (0.4-17.0)	1.9 (-1.8-5.6)	10.2 (0.6-19.9)	0	0
married/living together	6.0 (5.4-6.7)	5.7 (4.7-6.7)	6.5 (5.7-7.3)	6.9 (5.4-8.5)	7.8 (6.7-8.9)	4.5 (3.2-5.7)	4.7 (3.7-5.8)
widowed	8.2 (6.6-9.8)	5.5 (-5.5-16.2)	8.3 (6.7-9.8)	9.3 (-8.1-26.7)	10.3 (8-12.5)	0	5.4 (3.7-7.2)
divorced/separated	11.0 (6.1-15.8)	8.1 (6.7-17.2)	12.9 (7.2-18.5)	11.1 (-1.1-23.4)	14.4 (7.9-20.8)	0	3.6 (-3.4-10.7)
**Occupation**							
unemployed	8.5 (6.8-10.2)	8.1 (4.8-11.4)	8.7 (6.8-10.5)	8.7 (4.5-12.8)	9.7 (7.3-12.0)	6.7 (1.6-11.8)	6.3 (3.8-8.8)
manual	4.9 (4.3-5.6)	4.1 (3.0-5.2)	5.6 (4.7-6.5)	3.7 (1.6-5.8)	6.9 (5.5-8.4)	4.2 (2.9-5.5)	4.6 (3.6-5.6)
trades	7.3 (6.0-8.5)	6.2 (4.4-8.0)	8.7 (7.1-10.4)	6.9 (4.7-9.1)	9.2 (7.4-10.9)	4.1 (0.8-7.4)	6.0 (1.9-10.1)
professional	7.0 (3.9-10.0)	7.0 (3.6-10.3)	7.1 (2.6-11.7)	7.7 (3.3-12.1)	7.6 (2.5-12.7)	5.5 (0.7-10.2)	4.3 (-4.1-12.7)
other	6.5 (1.6-11.4)	3.7 (-0.3-7.6)	30.1 (13.2-47.1)	6.0 (-1.2-13.3)	39.1 (17.7-60.5)	1.4 (-1.5-4.3)	9.0 (-6.1-24.1)
retired/old age	9.6 (5.5-13.8)	11.4 (4.0-18.8)	8.8 (3.9-13.6)	14.4 (4.6-24.3)	13.5 (4.6-24.3)	13.5 (5.1-21.8)	3.1 (-0.3-6.5)
**BMI**							
Underweight	4.0 (2.1-5.9)	4.6 (1.7-7.4)	3.2 (1.3-5.0)	5.5 (-0.2-11.2)	5.1 (1.1-9.2)	3.9 (1.1-6.8)	2.0 (2.7-3.7)
Normal	4.9 (4.1-5.7)	4.5 (3.4-5.6)	5.4 (4.5-6.3)	5.2 (3.4-6.9)	7.0 (5.5-8.4)	4.0 (2.6-5.3)	3.9 (2.8-5.0)
Overweight	7.8 (6.5-9.1)	8.2 (5.8-10.6)	7.5 (6.1-8.9)	9.5 (6.2-12.9)	7.9 (6.1-9.7)	6.2 (2.9-9.4)	6.6 (4.6-8.7)
Obese	10.4 (8.5-12.2)	8.7 (2.9-14.5)	10.7 (8.9-12.5)	10.4 (2.2-18.6)	12.0 (9.8-14.2)	6.3 (-1.1-13.8)	6.7 (4.0-9.4)
**Family history of hypertension**[a]							
No	5.1 (4.3-5.9)	5.2 (3.9-6.5)	4.9 (4.1-5.8)	6.0 (4.1-7.9)	5.4 (4.3-6.5)	4.2 (2.6-5.8)	4.3 (3.0-5.6)
Yes	7.5 (6.5-8.5)	6.0 (4.4-7.5)	8.9 (7.7-10.0)	7.4 (4.8-10.1)	11.2 (9.6-12.9)	4.8 (2.9-6.7)	5.7 (4.3-7.0)
Don't know	6.4 (4.9-7.9)	5.6 (3.4-7.7)	7.2 (5.3-9.0)	8.5 (4.7-12.3)	10.1 (7.2-13.0)	3.3 (1.0-5.6)	3.9 (1.9-5.9)
**Alcohol consumption**[b]							
Never	6.3 (5.7-6.9)	5.6 (4.7-6.6)	7.0 (6.3-7.7)	6.8 (5.4-8.3)	8.5 (7.5-9.6)	4.4 (3.1-5.6)	4.9 (4.0-5.8)
Ever	4.1 (-1.3-9.5)	2.6 (-2.2-7.4)	8.4 (-7.6-24.4)	4.2 (-3.1-11.5)	27.4 (-4.4-59.3)	0	0

**Smoking status[c]**							
Current smoker	2.8 (1.3-4.3)	2.9 (1.4-4.3)	0	3.3 (1.0-5.5)	0	2.4 (0.4-4.4)	0
Never smoked	6.7 (6.0-7.3)	6.1 (4.9-7.3)	7.0 (4.9-7.3)	7.4 (5.6-9.2)	8.6 (7.6-9.6)	4.9 (3.2-6.5)	4.9 (4.0-5.7)
Previous smoker	6.5 (4.0-9.1)	6.6 (4.0-9.1)	0	8.7 (4.6-12.8)	0	4.1 (1.2-7.1)	0
**Wealth quintile**							
1 (Poorest)	4.8 (3.3-6.4)	3.3 (0.7-5.8)	6.8 (4.9-8.7)	2.1 (-1.9-6.1)	8.4 (0.2-16.5)	3.4 (0.6-6.1)	6.7 (4.7-8.7)
2	3.2 (2.1-4.4)	2.7 (1.2-4.2)	3.9 (2.4-5.5)	2.2 (-2.1-6.4)	5.9 (1.9-9.9)	2.8 (1.2-4.4)	3.5 (1.8-5.1)
3	5.4 (4.2-6.5)	5.8 (3.9-7.6)	4.9 (3.8-6.1)	4.2 (0.8-7.6)	4.3 (1.7-7.0)	6.1 (4.0-8.2)	5.1 (3.8-6.3)
4	5.7 (4.5-6.8)	4.9 (2.9-6.9)	6.4 (5.1-7.7)	5.6 (3.1-8.0)	6.9 (5.4-8.4)	2.8 (0.7-4.9)	4.1 (2.1-6.1)
5 (richest)	9.8 (8.5-11.2)	9.0 (6.6-11.3)	10.5 (9.1-11.9)	9.0 (6.6-11.3)	10.5 (9.1-11.9)	0	0

[a] self-report; [b] self-report of any alcohol consumption in the past 12 months; [c] self-reported tobacco use
Abbreviations: M=men; W=women

Supplementary Table 5: Age and sex-standardised prevalence (95% CI) of obesity by selected socio-demographic characteristics and risk factors weighted for cluster size

	Total			Urban		Rural	
	All	M	W	M	W	M	W
**All**	12.0 (11.1-12.9)	3.9 (3.0-4.8)	20.2 (18.8-21.5)	4.5 (3.2-5.8)	25.8 (23.8-27.7)	3.3 (2.0-4.6)	12.3 (10.6-14.0)
**Age categories**							
35-44	12.0 (10.8-13.2)	3.2 (2.0-4.5)	20.4 (18.7-22.1)	4.1 (2.0-6.2)	25.9 (23.5-28.4)	2.4 (0.9-3.8)	12.7 (10.5-14.9)
45-54	13.0 (11.4-14.7)	3.7 (1.9-5.4)	22.7 (20.0-25.3)	3.4 (0.9-5.8)	28.9 (25.1-32.7)	4.0 (1.4-6.5)	13.3 (10.3-16.4)
55-64	13.4 (11.5-15.3)	5.7 (3.4-8.1)	21.9 (18.9-24.9)	6.1 (3.1-9.1)	27.1 (22.7-31.5)	5.3 (1.6-8.9)	14.0 (10.3-17.8)
65-74	10.8 (8.6-13.0)	5.9 (3.5-8.4)	16.1 (12.7-19.5)	7.7 (4.0-11.4)	21.7 (16.5-27.0)	3.9 (0.9-7.0)	9.7 (5.9-13.5)
75-84	6.0 (3.4-8.5)	1.9 (-0.2-4.0)	9.5 (5.2-13.8)	2.5 (-0.9-6.0)	12.5 (5.7-19.3)	1.3 (-1.2-3.8)	6.0 (1.3-10.7)
85+	1.5 (-1.4-4.4)	0	2.7 (-2.4-7.9)	0	4.0 (-3.5-11.5)	0	0
**Level of education attained**							
Pre-school/no school	12.0 (10.0-14.0)	3.3 (1.4-5.2)	18.2 (15.3-21.0)	3.3 (0.8-5.8)	23.3 (19.6-27.0)	3.3 (0.5-6.2)	10.4 (7.2-13.7)
Primary	16.7 (14.2-19.3)	3.4 (0.9-5.9)	31.2 (27.3-35.2)	4.3 (0.4-8.3)	36..2 (31.6-40.9)	2.3 (-0.4-5.1)	16.1 (10.2-21.9)
Secondary/ vocational	11.6 (9.8-13.4)	3.0 (1.4-4.7)	29.5 (26.1-33.0)	3.4 (1.2-5.6)	32.4 (28.5-36.4)	2.3 (-0.1-4.8)	16.8 (11.0-22.7)
Higher	11.9 (8.0-15.9)	6.7 (2.8-10.6)	36.6 (27.5-45.8)	6.5 (2.3-10.8)	37.0 (27.3-46.6)	7.5 (-2.7-17.8)	32.7 (6.1-59.2)
Don't know/other	15.8 (9.9-21.8)	4.1 (-3.7-11.9)	20.3 (13.0-27.6)	0	21.0 (10.6-31.4)	4.8 (-4.3-13.9)	19.8 (9.9-29.8)
non-formal/Quranic	11.0 (9.8-12.2)	4.2 (2.8-5.6)	16.4 (14.8-18.0)	5.4 (3.1-7.7)	21.1 (18.7-23.5)	3.5 (1.8-5.2)	11.7 (9.7-13.7)
**Ethnicity**							
Mandinka	13.6 (12.0-15.2)	5.2 (3.3-7.2)	20.8 (18.7-22.9)	4.6 (2.5-6.7)	24.7 (22.1-27.4)	6.2 (2.4-9.9)	13.3 (10.5-16.0)
Wollof	10.4 (8.2-12.6)	2.8 (1.2-4.3)	19.0 (15.2-22.8)	3.5 (0.6-6.3)	29.9 (24.1-35.7)	2.4 (0.5-4.3)	9.9 (6.7-13.1)
Jola/Karoninka	14.0 (11.5-16.5)	3.2 (0.9-5.5)	23.9 (20.4-27.5)	4.5 (0.9-8.2)	27.8 (23.7-31.9)	1.5 (-0.4-3.5)	15.2 (9.8-20.7)
Fula/Tukulor/Lorobo	8.7 (7.0-10.3)	3.3 (1.8-4.7)	16.0 (13.3-18.8)	5.4 (2.4-8.3)	21.5 (17.7-25.3)	1.7 (0.4-2.9)	11.0 (7.3-14.6)
Sarahuleh	10.4 (7.8-13.0)	2.2 (-0.003-4.3)	16.9 (12.8-21.1)	2.0 (-0.9-4.9)	24.3 (17.6-30.9)	2.3 (-0.7-5.3)	11.7 (7.1-16.3)
Others	15.9 (12.9-18.9)	4.8 (1.9-7.7)	27.6 (22.5-32.6)	4.3 (0.4-8.3)	31.5 (25.6-37.3)	5.7 (1.8-9.6)	17.8 (9.4-26.2)
**Marital status**							
never married	7.7 (3.0-12.3)	3.7 (-0.6-8.0)	29.8 (16.7-43.0)	5.5 (-0.8-11.8)	29.3 (14.8-43.7)	0	33.6 (3.0-64.2)
married/living together	11.4 (10.5-12.3)	3.9 (2.9-4.8)	20.5 (19.0-21.9)	4.4 (3.1-5.8)	26.3 (24.3-8.3)	3.3 (2.0-4.6)	12.7 (10.9-14.6)
widowed	16.3 (13.8-18.8)	10.3 (-6.5-27.2)	16.5 (13.9-18.9)	0	20.7 (17.1-24.4)	23.3 (-4.2-50.8)	10.1 (7.2-13.0)
divorced/separated	25.0 (17.8-32.3)	3.7 (-3.5-10.9)	38.7 (29.8-47.6)	5.3 (-4.8-15.3)	42.3 (32.5-52.0)	0	16.0 (5.2-31.5)
**Occupation**							
unemployed	13.6 (11.3-15.9)	3.6 (1.4-5.9)	18.7 (15.7-21.8)	3.9 (1.2-6.6)	21.8 (17.9-25.6)	2.8 (-1.2-6.9)	11.8 (6.9-16.5)
manual	10.3 (9.2-11.5)	3.2 (1.8-4.5)	15.6 (14.2-17.1)	3.6 (1.3-6.0)	21.6 (19.2-24.0)	3.0 (1.4-4.6)	11.1 (9.5-12.8)
trades	16.3 (14.5-18.0)	5.0 (3.3-6.7)	31.9 (28.9-34.8)	5.1 (3.1-7.2)	32.5 (29.2-35.7)	4.5 (1.8-7.2)	28.0 (21.9-34.1)
professional	8.1 (5.6-10.6)	4.5 (2.0-6.9)	3.1 (23.2-39.6)	4.8 (1.6-8.0)	33.7 (24.7-42.7)	3.8 (-0.04-7.7)	16.9 (0.4-33.3)
other	5.0 (1.9-8.1)	2.6 (-0.3-5.6)	25.8 (6.5-45.2)	2.1 (-1.9-6.2)	31.5 (7.2-55.8)	3.0 (-1.1-7.2)	11.2 (-10.9-33.3)
retired/old age	7.4 (3.8-11.0)	0	11.1 (6.0-16.3)	0	16.8 (9.0-24.6)	0	3.7 (-1.5-9.0)
**Family history of hypertension**[a]							
No	10.4 (9.3-11.6)	3.7 (2.5-4.9)	18.3 (16.5-20.2)	5.0 (3.2-6.9)	22.5 (20.1-25.0)	2.1 (0.8-3.3)	11.9 (9.5-14.4)
Yes	14.7 (13.3-16.1)	4.3 (2.7-5.8)	23.6 (21.6-25.6)	3.8 (1.8-5.8)	30.4 (27.7-33.2)	4.7 (2.4-7.0)	14.2 (11.8-16.6)
Don't know	9.0 (7.0-11.0)	3.3 (1.2-5.4)	14.6 (11.5-17.8)	3.9 (0.3-7.6)	20.6 (15.8-25.4)	2.9 (0.5-5.3)	7.5 (4.6-10.4)
**Alcohol consumption**[b]							
Never	12.1 (11.1-13.0)	3.9 (3.0-4.8)	20.2 (3.0-21.5)	4.5 (3.1-5.8)	25.8 (23.8-27.7)	3.3 (2.0-4.6)	12.3 (10.6-14.0)
Ever	8.0 (3.0-13.0)	4.5 (-0.9-9.8)	19.6 (10.5-28.7)	4.5 (-3.1-12.0)	23.9 (2.8-44.9)	4.5 (-1.9-10.9)	17.9 (8.4-27.4)
**Smoking status**[c]							
Current smoker	1.8 (0.5-3.0)	1.8 (0.5-3.0)	0	0.4 (-0.1-0.9)	0	3.3 (0.8-5.8)	0
Never smoked	14.0 (12.9-15.0)	4.4 (3.3-5.6)	20.2 (18.8-21.6)	5.6 (3.8-7.5)	25.8 (23.9-27.7)	3.3 (1.9-4.7)	12.3 (10.6-14.0)
Previous smoker	4.3 (2.2-6.5)	4.4 (2.2-6.5)	0	5.1 (1.9-8.3)	0	3.5 (0.7-6.4)	0

**Wealth quintile**							
1 (poorest)	7.7 (5.6-9.7)	2.5 (0.5-4.5)	14.0 (10.1-17.8)	0	13.7 (2.6-24.7)	2.7 (0.5-5.0)	14.0 (9.9-18.0)
2	7.0 (5.5-8.5)	2.3 (0.9-3.7)	13.1 (10.2-15.9)	0.8 (-0.7-2.3)	22.5 (15.5-29.4)	2.6 (0.9-4.3)	10.7 (7.8-13.6)
3	8.4 (7.0-9.8)	4.1 (2.3-5.9)	13.2 (11.0-15.3)	3.8 (-0.5-8.1)	18.0 (12.4-23.5)	4.2 (2.2-6.2)	12.1 (9.8-14.3)
4	13.6 (11.8-15.5)	4.3 (2.3-6.2)	22.5 (19.9-25.2)	4.8 (2.5-7.2)	24.3 (21.3-27.4)	2.5 (-0.4-5.5)	14.5 (11.1-17.8)
5 (richest)	18.2 (16.2-20.1)	5.0 (3.0-7.1)	28.3 (25.9-30.7)	5.0 (3.0-7.1)	28.3 (25.9-30.7)	0	0
**Diabetes status**[d]							
No	11.5 (10.6-12.4)	3.8 (2.8-4.7)	19.4 (18.0-20.7)	4.3 (3.0-5.6)	24.8 (22.8-26.8)	3.2 (1.9-4.6)	12.1 (10.4-13.8)
Yes	20.1 (16.6-23.6)	6.2 (2.1-10.3)	31.1 (26.7-35.5)	7.1 (1.3-13.0)	36.5 (31.1-41.8)	4.7 (-0.4-9.9)	17.6 (10.8-24.3)

[a] self-report; [b] self-report of any alcohol consumption in the past 12 months; [c] self-reported tobacco use; [d] defined as a fasting blood glucose level ≥7mmol/L or random blood glucose of ≥11.1mmol/L and/or self-reported history of health personnel diagnosis of diabetes and/or currently receiving treatment for diabetes
Abbreviations: M=men; W=women

Supplementary Table 6: Age and sex-standardised prevalence (95% CI) of multimorbidity by selected socio-demographic characteristics and risk factors weighted for cluster size

	Total			Urban		Rural	
	**All**	**M**	**W**	**M**	**W**	**M**	**W**
All	10.7 (9.9-11.5)	5.5 (4.6-6.4)	15.9 (14.7-17.2)	5.8 (4.6-7.1)	19.7 (18.0-21.5)	5.1 (3.8-6.4)	10.6 (9.0-12.1)
**Age categories**							
35-44	6.9 (6.0-7.6)	1.9 (0.9-2.8)	11.6 (10.3-12.8)	1.5 (0.3-2.7)	14.2 (12.4-15.9)	2.2 (0.8-3.7)	8.0 (6.2-9.8)
45-54	12.9 (11.4-14.4)	6.3 (4.4-8.2)	19.8 (17.3-22.2)	6.1 (3.5-8.7)	24.2 (20.7-27.6)	6.4 (3.7-9.2)	13.1 (10..0-16.2)
55-64	16.4 (14.2-18.5)	10.7 (7.6-13.8)	22.7 (19.6-25.7)	12.7 (8.2-17.3)	28.2 (23.9-32.5)	8.3 (4.3-12.2)	14.4 (4.3-12.2)
65-74	15.1 (12.6-17.6)	11.7 (8.7-14.8)	18.8 (15.1-22.6)	12.7 (8.5-16.9)	24.4 (18.4-30.3)	10.7 (6.3-15.1)	12.5 (8.5-16.6)
75-84	9.5 (6.4-12.7)	5.2 (1.7-8.8)	13.3 (8.4-18.1)	6.5 (0.9-12.1)	18.0 (10.2-25.7)	4.0 (-0.4-8.4)	7.8 (2.6-13.0)
85+	7.2 (1.1-13.4)	5.0 (-1.8-11.7)	9.1 (-0.6-18.7)	7.3 (-6.5-21.1)	8.5 (-2.5-19.5)	3.6 (-3.4-10.6)	10.2 (-8.7-29.0)
**Level of education attained**							
Pre-school/no school	12.2 (10.2-14.1)	7.0 (4.5-9.4)	15.8 (13.3-18.4)	9.4 (5.4-13.4)	20.2 (16.8-23.5)	4.8 (1.8-7.8)	9.4 (6.3-12.4)
Primary	12.6 (10.4-14.8)	3.8 (1.5-6.1)	22.2 (18.7-25.7)	4.5 (1.0-8.0)	25.2 (20.8-29.5)	3.0 (0.1-5.9)	13.2 (8.5-17.9)
Secondary/ vocational	8.7 (7.1-10.3)	4.2 (2.5-5.9)	18.1 (15.1-21.1)	4.0 (2.0-5.9)	19.9 (16.5-23.4)	4.8 (1.4-8.2)	10.0 (5.1-14.8)
Higher	9.7 (6.3-13.1)	6.6 (2.9-10.4)	24.2 (14.9-33.4)	6.5 (2.4-10.6)	24.6 (14.8-34.4)	7.3 (-2.1-16.7)	19.0 (-3.1-41.2)
Don't know/other	14.9 (8.8-21.1)	9.4 (-1.6-20.4)	17.0 (10.4-23.6)	0	22.9 (12.1-33.7)	11.0 (2.0-24.0)	13.1 (5.3-20.9)
non-formal/Quranic	10.4 (9.3-11.5)	5.8 (4.5-7.1)	14.0 (12.6-15.5)	6.4 (4.2-8.6)	17.5 (15.4-19.7)	5.4 (3.8-7.0)	10.6 (8.6-12.5)
**Ethnicity**							
Mandinka	11.7 (10.4-13.1)	6.2 (4.6-7.9)	16.5 (14.7-18.3)	5.3 (3.4-7.3)	19.1 (16.8-21.5)	7.5 (4.6-10.4)	11.4 (8.8-14.0)
Wollof	10.2 (8.4-12.0)	5.6 (3.6-7.6)	15.4 (12.5-18.4)	4.9 (1.8-8.0)	23.2 (18.4-27.9)	6.0 (3.4-8.6)	8.9 (6.1-11.8)
Jola/Karoninka	11.0 (8.6-13.3)	4.2 (2.0-6.5)	17.2 (13.9-20.4)	6.0 (2.5-9.4)	19.6 (15.6-23.6)	2.0 (-0.3-4.3)	11.7 (6.7-16.7)
Fula/Tukulor/Lorobo	7.8 (6.3-9.3)	4.4 (2.9-5.9)	12.5 (10.1-14.8)	6.4 (3.4-9.3)	15.0 (1.4-4.4)	2.9 (1.4-4.4)	10.1 (6.8-13.3)
Sarahuleh	10.7 (8.0-13.4)	6.5 (2.3-10.8)	14.0 (10.4-17.7)	8.4 (-0.1-16.8)	17.9 (12.2-23.6)	5.3 (0.8-9.8)	11.2 (6.7-15.8)
Others	14.5 (11.3-17.7)	6.2 (1.7-9.5)	23.3 (18.2-28.3)	6.1 (1.3-10.8)	28.7 (22.6-34.7)	6.3 (2.3-10.3)	9.6 (5.1-14.2)
**Marital status**							
never married	2.2 (0.3-4.2)	0.8 (-0.8-2.3)	10.5 (1.3-19.6)	1.1 (-1.1-3.4)	10.1 (0.2-20.1)	0.00	12.6 (-10.8-35.9)
married/living together	9.9 (9.1-10.7)	5.7 (4.7-6.6)	15.0 (13.7-16.3)	6.1 (4.7-7.4)	18.5 (16.7-20.4)	5.3 (4.0-6.6)	10.3 (8.6-11.9)
widowed	18.3 (15.6-21.0)	7.4 (-6.8-21.6)	18.6 (15.9-21.3)	13.3 (-11.2-37.8)	23.1 (19.3-26.9)	0.00	11.8 (8.4-15.2)
divorced/separated	18.2 (13.3-23.2)	2.7 (-2.7-8.1)	28.2 (21.6-34.9)	3.9 (-3.7-11.4)	30.6 (23.4-37.8)	0.00	13.1 (-1.4-27.6)
**Occupation**							
unemployed	16.1 (13.5-18.7)	10.0 (6.0-14.0)	19.2 (16.0-22.4)	10.9 (5.8-16.0)	21.7 (17.6-25.7)	7.7 (1.9-13.5)	13.5 (8.9-18.2)
manual	9.1 (8.1-10.1)	4.6 (3.4-5.8)	12.4 (11.1-13.7)	3.8 (1.8-5.9)	16.3 (14.1-18.5)	4.9 (3.5-6.3)	9.5 (7.9-11.1)
trades	12.4 (10.9-13.9)	5.4 (3.8-7.0)	22.0 (19.4-24.6)	5.6 (3.8-7.5)	22.1 (19.3-24.9)	4.7 (1.5-7.8)	21.4 (15.2-27.7)
professional	7.4 (4.9-9.9)	5.6 (3.0-8.3)	18.6 (11.6-25.7)	5.0 (1.9-8.1)	20.5 (12.7-28.4)	6.9 (1.7-12.0)	6.2 (-5.6-18.1)
other	8.1 (2.7-13.4)	4.9 (0.7-9.0)	35.8 (13.7-58.0)	5.3 (-2.1-12.7)	49.7 (24.3-75.1)	4.5 (0.1-8.8)	0.00
retired/old age	13.6 (8.6-18.6)	9.9 (2.6-17.2)	15.4 (9.4-21.5)	12.1 (2.4-21.8)	24.3 (15.1-33.4)	5.2 (-4.9-15.3)	3.6 (-1.6-8.9)
**Alcohol consumption**[a]							
Never	10.7 (9.9-11.5)	5.5 (4.6-6.4)	15.9 (14.7-17.2)	5.9 (4.6-7.1)	19.7 (18.0-21.4)	5.1 (3.8-6.4)	10.6 (9.0-12.1)
Ever	7.3 (2.2-12.3)	4.5 (-0.8-9.8)	16.5 (2.6-30.3)	4.5 (-3.1-12.0)	34.5 (14.5-54.5)	4.5 (-1.9-10.9)	9.3 (-0.7-19.4)
**Smoking status**[b]							
Current smoker	2.2 (1.0-3.4)	2.2 (1.0-3.4)	0.00	1.6 (0.3-3.0)	0.00	2.8 (0.8-4.9)	0.00
Never smoked	12.1 (11.2-13.0)	6.2 (5.0-7.3)	16.0 (14.7-17.2)	6.5 (4.9-8.1)	19.8 (18.0-21.5)	5.8 (4.2-7.4)	10.6 (9.1-12.1)
Previous smoker	6.8 (4.1-9.4)	6.8 (4.1-9.4)	0.00	8.5 (4.4-12.7)	0.00	4.8 (1.8-7.7)	0.00
**Wealth quintile**							
1 (poorest)	7.7 (5.6-9.8)	2.7 (0.5-4.9)	13.8 (10.1-17.5)	2.2 (-2.0-6.3)	10.7 (1.2-20.2)	2.8 (0.4-5.2)	14.1 (10.1-18.0)
2	6.2 (4.8-7.7)	3.7 (1.8-5.6)	9.5 (7.1-11.9)	0.8 (-0.7-2.3)	16.5 (9.9-23.1)	4.4 (2.1-6.8)	7.7 (5.2-10.1)

3	8.6 (7.1-10.0)	6.3 (4.4-8.3)	11.1 (9.2-13.0)	3.3 (0.4-6.3)	13.5 (8.7-18.3)	7.0 (4.7-9.3)	10.5 (8.5-12.6)
4	10.5 (9.1-12.0)	4.9 (3.1-6.6)	15.9 (13.6-18.2)	5.3 (3.1-7.4)	16.8 (14.2-19.5)	3.5 (1.4-5.7)	11.7 (8.1-15.2)
5 (richest)	16.3 (14.5-18.0)	7.6 (5.6-9.5)	22.9 (20.6-25.2)	7.6 (5.6-9.5)	22.9 (20.6-25.2)	0.00	0.00

[a] self-report of any alcohol consumption in the past 12 months; [b] self-reported tobacco use
Abbreviations: M=men; W=women

Supplementary Table 7: Association of risk factors with hypertension and diabetes in the study population, adjusted for non-modifiable and contextual factors*

	Hypertension		Diabetes	
Variable	Men	Women	Men	Women
**Residence**				
Urban	1	1	1	1
Rural	1.29 (0.96-1.74)	1.04 (0.85-1.28)	1.23 (0.72-2.09)	0.81 (0.58-1.15)
**Age group**				
35-44	1	1	1	1
45-54	1.83 (1.46-2.30)	2.21 (1.94-2.52)	3.24 (2.02-6.29)	1.74 (1.32-2.29)
55-64	4.17 (3.30-5.26)	3.29 (2.78-3.90)	3.56 (2.02-6.29)	1.99 (1.46-2.73)
65-74	5.72 (4.40-7.45)	4.78 (3.66-6.25)	3.61 (1.94-6.73)	2.11 (1.35-3.30)
75-84	5.56 (3.77-8.19)	4.82 (3.40-6.83)	2.51 (1.12-5.62)	2.02 (1.12-3.67)
85+	4.47 (2.40-8.32)	5.12 (2.48-10.57)	1.69 (0.42-6.69)	0.71 (0.14-3.56)
**Level of education attained**				
Pre-school/no school	1	1	1	1
Primary	0.86 (0.60-1.26)	1.01 (0.80-1.28)	0.54 (0.25-1.17)	1.33 (0.91-1.95)
Secondary/vocational)	0.92 (0.67-1.26)	0.95 (0.75-1.21)	0.85 (0.47-1.54)	1.09 (0.73-1.65)
Higher	0.87 (0.55-1.37)	0.93 (0.55-1.56)	1.40 (0.59-3.31)	0.84 (0.28-2.48)
Don't know/other	1.53 (0.67-3.51)	1.19 (0.75-1.89)	1.56 (0.40-6.12)	0.70 (0.30-1.61)
non-formal/Quranic	1.07 (0.82-1.40)	0.95 (0.81-1.12)	0.74 (0.46-1.19)	0.80 (0.59-1.09)
**Ethnicity**				
Mandinka	1	1	1	1
Wollof	0.85 (0.64-1.13)	1.09 (0.90-1.32)	1.16 (0.71-1.90)	1.40 (1.03-1.90)
Jola/Karoninka	1.02 (0.74-1.41)	0.78 (0.65-0.94)	0.73 (0.40-1.36)	0.74 (0.50-1.09)
Fula/Tukulor/Lorobo	0.94 (0.73-1.21)	1.17 (0.98-1.39)	1.04 (0.64-1.70)	0.95 (0.67-1.35)
Sarahuleh	1.60 (1.10-2.34)	1.53 (1.23-1.91)	1.02 (0.44-2.38)	0.97 (0.64-1.48)
Others	0.94 (0.61-1.45)	1.21 (0.93-1.58)	0.59 (0.27-1.28)	1.43 (0.95-2.17)
**Marital status**				
never married	1	1	1	1
married/living together	1.20 (0.71-2.04)	1.35 (0.74-2.48)	3.59 (0.54-23.93)	1.00 (0.33-2.99)
widowed	1.64 (0.42-6.46)	2.10 (1.13-3.90)	3.72 (0.21-69.95)	1.01 (0.33-3.08)
divorced/separated	1.43 (0.54-3.77)	1.48 (0.76-2.88)	5.63 (0.53-59.56)	1.46 (0.44-4.80)
**Occupation**				
unemployed	1.45 (1.04-2.02)	1.41 (1.14-0.75)	1	1
manual	1	1	0.60 (0.33-1.09)	0.79 (0.57-1.10)
trades	0.89 (0.70-1.12)	1.07 (0.92-1.25)	0.97 (0.54-1.74)	0.98 (0.69-1.40)
professional	0.99 (0.70-1.12)	1.38 (0.90-2.10)	0.91 (0.43-1.91)	0.81 (0.33-1.97)
other	0.76 (0.42-1.37)	2.21 (0.72-6.74)	0.63 (0.18-2.21)	4.73 (2.02-11.07)
retired/old age	1.67 (0.86-3.25)	1.67 (1.01-2.74)	1.26 (0.52-3.06)	0.93 (0.45-4.80)
**Wealth quintile**				
1 (poorest)	1	1	1	1
2	1.17 (0.81-1.69)	0.71 (0.55-0.92)	0.93 (0.37-2.33)	0.54 (0.33-0.88)
3	1.01 (0.71-1.43)	0.89 (0.69-1.13)	1.88 (0.78-4.56)	0.67 (0.46-0.99)
4	1.36 (0.91-2.03)	0.78 (0.59-1.05)	1.55 (0.62-3.86)	0.71 (0.47-1.08)
5 (richest)	1.28 (0.83-1.98)	0.91 (0.68-1.22)	2.98 (1.17-7.56)	1.09 (0.71-1.69)

**BMI**				
Mean (SD)	1.09 (1.06-1.11)	1.08 (1.06-1.09)	1.05 (1.01-1.10)	1.04 (1.01-1.08)
Underweight	0.61 (0.44-0.85)	0.65 (0.49-0.85)	1.10 (0.55-2.21)	0.56 (0.30-1.07)
Normal	1	1	1	1
Overweight	1.68 (1.32-2.14)	1.47 (1.28-1.69)	1.68 (1.11-2.52)	1.26 (0.95-1.66)
Obese	1.86 (1.21-2.84)	2.58 (2.23-2.98)	1.58 (0.66-3.79)	1.69 (1.30-2.20)
**Alcohol consumption**[a]				
Never	1	1	1	1
Ever	2.01 (1.08-3.76)	0.72 (0.47-1.12)	0.44 (0.09-2.18)	0.94 (0.12-7.51)
**Smoking status**[b]				
Current smoker	0.71 (0.55-0.90)	0.57 (0.12-2.62)	0.55 (0.30-1.02)	empty
Never smoked	1	1	1	empty
Previous smoker	1.09 (0.82-1.43)	1 (empty)	1.08 (0.67-1.73)	empty

* Adjusted for age, ethnicity, education, residence, wealth quintile, occupation, marital status

Data are in OR (95% confidence interval); [a] self-report of any alcohol consumption in the past 12 months; [b]self-reported smoking

Supplementary Table 8: Association of risk factors with obesity in the study population, adjusted for non-modifiable and contextual factors*

Variable	Men	Women
**Residence**		
Urban	1	1
Rural	1.26 (0.63-2.52)	0.71 (0.55-0.92)
**Age group**		
35-44	1	1
45-54	1.16 (0.62-2.17)	1.25 (1.04-1.49)
55-64	1.88 (1.01-3.49)	1.30 (1.03-1.63)
65-74	2.14 (1.06-4.33)	1.00 (0.75-1.35)
75-84	0.79 (0.21-2.94)	0.56 (0.32-0.99)
85+	empty	0.15 (0.21-1.00)
**Level of education attained**		
Pre-school/no school	1	1
Primary	0.95 (0.35-2.55)	1.59 (1.23-2.05)
Secondary/vocational)	0.83 (0.35-1.97)	1.35 (1.06-1.72)
Higher	1.95 (0.65-5.83)	1.66 (1.01-2.73)
Don't know/other	1.49 (0.17-12.81)	1.20 (0.73-1.99)
non-formal/Quranic	1.32 (0.67-2.60)	0.90 (0.73-1.11)
**Ethnicity**		
Mandinka	1	1
Wollof	0.51 (0.24-1.08)	0.94 (0.74-1.19)
Jola/Karoninka	0.59 (0.25-1.39)	1.12 (0.90-1.40)
Fula/Tukulor/Lorobo	0.61 (0.31-1.15)	0.83 (0.66-1.05)
Sarahuleh	0.39 (0.13-1.13)	1.02 (0.76-1.37)
Others	0.93 (0.44-1.97)	1.19 (0.91-1.55)
**Marital status**		
never married	1	1
married/living together	0.93 (0.26-3.32)	1.02 (0.54-1.93)
widowed	4.44 (0.34-57.20)	0.93 (0.47-1.82)
divorced/separated	1.03 (0.09-11.35)	1.73 (0.83-3.60)
**Occupation**		
unemployed	1	1
manual	1.16 (0.48-2.85)	0.83 (0.65-1.05)
trades	1.99 (0.87-4.57)	1.48 (1.15-1.89)
professional	1.31 (0.43-3.99)	1.07 (0.64-1.79)
other	1.11 (0.26-4.70)	1.23 (0.44-3.44)
retired/old age	empty	0.93 (0.52-1.65)
**Wealth quintile**		
1 (poorest)	1	1
2	1.00 (0.37-2.72)	0.91 (0.62-1.33)
3	1.77 (0.73-4.33)	0.89 (0.62-1.28)
4	1.85 (0.70-4.93)	1.11 (0.76-1.60)
5 (richest)	1.97 (0.70-5.55)	1.30 (0.88-1.92)
**Alcohol consumption**[a]		
Never	1	1
Ever	1.00 (0.26-3.80)	1.14 (0.64-2.01)
**Smoking status**[b]		
Current smoker	0.39 (0.19-0.82)	empty
Never smoked	1	empty
Previous smoker	0.98 (0.55-1.74)	empty

* Adjusted for age, ethnicity, education, residence, wealth quintile, occupation, marital status

Data are in OR (95% confidence interval); [a] self-report of any alcohol consumption in the past 12 months; [b] self-reported smoking
