# Supplementary material for: Prevalence of hypertension, diabetes, obesity, multimorbidity, and related risk factors among adult Gambians: a cross-sectional nationwide study
Source: Lancet Glob Health. Author manuscript; Available in PMC 2025 Oct 18. (PMC7618049; doi:10.1016/S2214-109X(23)00508-9)
Supplement: Supplementary appendix 2 [file EMS208202-supplement-Supplementary_appendix_2.pdf]

THE LANCET
Global Health

Supplementary appendix 2

This Equitable Partnership Declaration (EPD) was submitted by the authors, and we reproduce it as supplied. It has not been peer reviewed. *The Lancet*'s editorial processes have not been applied to the EPD.

Supplement to: Jobe M, Mactaggart I, Bell S, et al. Prevalence of hypertension, diabetes, obesity, multimorbidity, and related risk factors among adult Gambians: a cross-sectional nationwide study. *Lancet Glob Health* 2024; **12:** e55–65.

Equitable Partnership Declaration questions

This Equitable Partnership Declaration is a statement being published online alongside papers at *The Lancet Global Health*, as a separate appendix, to allow researchers to describe how their work engages with researchers, communities, and environments in the countries of study. This is part of our broader goal to decolonise global health, handing control and leadership of research to academics and clinicians who are based in the regions of study, and to affected communities.

Please answer all questions with as much detail as possible, noting that all included information will be published open-access and it will be freely available online to all who wish to read it. If a question does not apply to your study, please state "Not applicable".

The format of and questions in this statement are currently in a pilot phase. Please email Dr Liam Messin (Liam.Messin@lancet.com; deputy editor) and Dr Kate McIntosh (Kate.McIntosh@lancet.com; senior editor) with any feedback, particularly if you find any questions unclear.

Researcher considerations

1. Please detail the involvement that researchers who are based in the region(s) of study had during a) study design; b) clinical study processes, such as processing blood samples, prescribing medication, or patient recruitment; c) data interpretation; and d) manuscript preparation, commenting on all aspects. If they were not involved in any of these aspects, please explain why.

This question is intended for international partnerships; if all your authors are based in the area of study, this question is not applicable.

This should include a thorough description of their leadership role(s) in the study. Are local researchers named in the author list or the acknowledgements, or are they not mentioned at all (and, if not, why)? Please also describe the involvement of early career researchers based in the location of the study. Some of this information might be repeated from the Contributors section in the manuscript. Note: we adhere to ICMJE authorship criteria when deciding who should be named on a paper.

**a) Study design:** The study was jointly led by two principal investigators, one of whom was Dr. Abba Hydara, a Gambian Ophthalmologist. The study was designed with the involvement of additional local investigators, including Dr. Modou Jobe, a Gambian cardiologist, and Mr. Nyakassi Sanyang, the Statistician General at the Gambia Bureau of Statistics (GBoS). This includes protocol development, design and development of CRFs, standard operating procedures for study activities, planning and implementation of data collection activities. These were conducted through regular online and face-to-face meetings involving local and non-locally based investigators.
**b) Clinical study processes:** The implementation of the study was led by a local principal investigator, Dr. Abba Hydara. He supervised all clinical aspects of the study with input from investigators based elsewhere. The clinical procedures, including standardization of diagnostic criteria, recruitment of field staff and study participants, collection and processing of blood samples and buccal swabs, anthropometric measurements were carried out by local staff. International field team members supported in ophthalmology.

**c) Data interpretation:** The data interpretation for this manuscript was led by a local researcher (Dr. Modou Jobe) with participation of other investigators.
**d) Manuscript preparation:** The draft of manuscript was prepared by one of the local investigators (Dr Modou Jobe). This was subsequently reviewed by other local and externally based investigators.

2. Were the data used in your study collected by authors named on the paper, or have they been extracted from a source such as a national survey? That is: is this a secondary analysis of data that were not collected by the authors of this paper. If the authors of this paper were not involved in data collection, how were data interpreted with sufficient contextual knowledge?

   The Lancet Global Health *believe contextual understanding is crucial for informed data analysis and interpretation.*

The primary data was collected by authors named on the paper. The data was therefore interpreted with sufficient contextual knowledge. The population data for sampling was sourced from the National Population Census of 2013 report by the GBoS.

3. How was funding used to remunerate and enhance the skills of researchers and institutions based in the area(s) of study? And how was funding used to improve research infrastructure in the area of study?

   Potentially effective investments into long-term skills and opportunities within institutions could include training or mentorship in analytical techniques and manuscript writing, opportunities to lead all or specific aspects of the study, financial remuneration rather than requiring volunteers, and other professional development and educational opportunities.

   Improvements to research infrastructure could be funding of extended trial designs (such as platform trials) and use of master protocols to enable these designs, establishment of long-term contracts for research staff, building research facilities, and local control of funding allocation.

Skills: The collaborating institutions in this project were the Sheikh Zayed Regional Eye Care Centre (SZRECC) and the National Eye Health Programme, both of the Ministry of Health of The Gambia, the MRC Unit The Gambia at London School of Hygiene and Tropical Medicine, and the GBoS. The staff implementing the clinical measurements are all health workers with little research experience prior to this project. The project has greatly exposed them to conducting research studies, especially in electronic data collection procedures. Furthermore, the staff have been trained on many aspects (using point-of-care devices) of the study which is outside their routine work. The overall research capacity of the local staff has been sufficiently elevated from this

research exposure, e.g., using the SLR Camera for high definition anterior segment images, the Remedio Fundus Camera for optic disc and macular images, the systematic administration of a research protocol in a field situation, repeated acquisition of near and far vision through refraction and anthropometric measures, administration of structured mental health questionnaires for anxiety and depression, and administration of the Washington Group questionnaires for disability assessment.

As most staff were employees of SZRECC/NEHP and GBoS, financial remuneration e.g. daily subsistence allowance, night allowance and telephone allowance (specific to the household listing teams made up of GBoS staff) etc. were paid in accordance with Gambia Government guidelines.

Research infrastructure:
The SZRECC is a public institution for **service delivery, training** and **research**. However, this project greatly enhanced the latter two through capacity building of staff and retention of equipment used during the project implementation.

4. How did you safeguard the researchers who implemented the study?

Please describe how you guaranteed safe working conditions for study staff, including provision of appropriate personal protective equipment, protection from violence, and prevention of overworking.

All procedures were conducted in accordance with Gambia government guidelines and Gambia Labour laws which cater for staff welfare, health & safety, employment rights and working conditions. The study was approved by the Gambia Government/MRC Joint Scientific and Ethics Committee.
Staff were given official survey identification cards which they wore throughout the fieldwork.
Each survey field team had a first aid kit with basic medications, including antimalarial agents as well as items for addressing simple cuts and injuries.
All Regional Health Teams were aware of the presence of the survey teams in their communities as well as local social and administrative authorities during the fieldwork.

Benefits to the communities and regions of study

5. How does the study address the research and policy priorities of its location?

How were the local priorities determined and then used to inform the research question? Who decided which priorities to take forward? Which elements of the study address those priorities?

This eye health and comorbidities survey was designed as a follow up to the Gambia National Eye Health Surveys of 1986 and 1996. The proposal was discussed with local researchers as well as with relevant authorities at the Ministry of Health. The local stakeholders agreed that it was relevant to include evaluation of non-communicable diseases and related risk factors. This against the backdrop that the most recent nationwide survey on non-communicable diseases was nearly a decade at the time of conducting this study. This paper provides an updated prevalence of hypertension, diabetes, obesity and multi-morbidity which is crucial to the implementation of country’s recently launched non-communicable diseases multi-sectoral action plan.

6. How will research products be shared in the community of study?

For instance, will you be providing written or oral layperson summaries for non-academic information sharing? Will study data be made available to institutions in the region(s) of study? The Lancet Global Health *encourages authors to translate the summary (abstract) into relevant languages after paper editing; do you intend to translate your summary?*

Research findings will be communicated to the public through the media teams at both the Medical Research Council Unit the Gambia and London School of Hygiene and Tropical Medicine, and through local newspapers to publicise relevant findings to the wider public through a press release. The illiterate members of the community would be reached through health-related radio programmes.

7. How were individuals, communities, and environments protected from harm?

a) How did you ensure that sensitive patient data was handled safely and respectfully? Was there any potential for stigma or discrimination against participants arising from any of the procedures or outcomes of the study?

The proposal was reviewed by the Joint MRC/Gambia Government Ethics Committee and the London School of Hygiene & Tropical Medicine Ethics Committee.

Study activities and procedures were explained in detail to all study participants and they were given options to consent to participate or decline participating in this study. During interviews, we encouraged participants to let us know and not to answer any questions that they are not comfortable with without giving any reasons.

We used clean, sterile phlebotomy equipment and local pressure applied to avoid post-puncture bleeding.

We ensured that participants' privacy and modesty was preserved as much as required.

Data was anonymized prior to the analyses such that no specific data sets can be traced back to individuals.

b) Might any of the tests be experienced as invasive or culturally insensitive?

No

c) How did you determine that work was sensitive to traditions, restrictions, and considerations of all cultural and religious groups in the study population?

Local investigators (led by Drs. Hydara and Jobe) were involved throughout every phase of the development of this research. Multiple local staff were involved in development of the field procedures and questionnaires. The proposals were scrutinised and approved by the MRCG Scientific Coordinating Committee and the Joint MRC/Gambia Government Ethics Committee both of which have numerous local experts.

d) *Were bio-waste and radioactive waste disposed of in accordance with local laws?*

Yes

e) *Were any structures built that would have impacted members of the community or the environment (such as handwashing facilities in a public space)? If so, how did you ensure that you had appropriate community buy-in?*

No

f) *How might the study have impacted existing health-care resources (such as staff workloads, use of equipment that is typically employed elsewhere, or reallocation of public funds)?*

This survey was conducted with the participation of staff of the Gambia Bureau of Statistics, Ministry of Health of The Gambia and Sheikh Zayed Regional Eye Care Centre. However, these staff were mainly seconded to the project during the data collection without hampering routine operations. The project procured equipment used in this study and did not disrupt routine clinical services.

8. Finally, please provide the title (e.g., Dr/Prof, Mr/Mrs/Ms/Mx), name, and email address of an author who can be contacted about this statement. This can be the corresponding author.

Name: Dr Modou Jobe
Email: Modou.Jobe@lshtm.ac.uk
